# Mortality and cardiovascular events in adults with kidney failure after major non-cardiac surgery: a population-based cohort study

**DOI:** 10.1186/s12882-021-02577-7

**Published:** 2021-11-04

**Authors:** Tyrone G. Harrison, Paul E. Ronksley, Matthew T. James, Shannon M. Ruzycki, Marcello Tonelli, Braden J. Manns, Kelly B. Zarnke, Deirdre McCaughey, Prism Schneider, James Wick, Brenda R. Hemmelgarn

**Affiliations:** 1 Department of Medicine, University of Calgary, Calgary, Alberta Canada; 2 Department of Community Health Sciences, University of Calgary, Calgary, Alberta Canada; 3 O’Brien Institute for Public Health, Cumming School of Medicine, University of Calgary, Calgary, Alberta Canada; 4 Libin Cardiovascular Institute, Cumming School of Medicine, University of Calgary, Calgary, Alberta Canada; 5 Department of Surgery, University of Calgary, Calgary, Alberta Canada; 6 Department of Medicine, University of Alberta, 2J2.01 Walter C. Mackenzie Health Sciences Centre, Edmonton, Alberta T6G 2R7 Canada

**Keywords:** Kidney failure, Perioperative, Major surgery, Outcomes, Cohort study

## Abstract

**Background:**

People with kidney failure have a high incidence of major surgery, though the risk of perioperative outcomes at a population-level is unknown. Our objective was to estimate the proportion of people with kidney failure that experience acute myocardial infarction (AMI) or death within 30 days of major non-cardiac surgery, based on surgery type.

**Methods:**

In this retrospective population-based cohort study, we used administrative health data to identify adults from Alberta, Canada with major surgery between April 12,005 and February 282,017 that had preoperative estimated glomerular filtration rates (eGFRs) < 15 mL/min/1.73m^2^ or received chronic dialysis. The index surgical procedure for each participant was categorized within one of fourteen surgical groupings based on Canadian Classification of Health Interventions (CCI) codes applied to hospitalization administrative datasets. We estimated the proportion of people that had AMI or died within 30 days of the index surgical procedure (with 95% confidence intervals [CIs]) following logistic regression, stratified by surgery type.

**Results:**

Overall, 3398 people had a major surgery (1905 hemodialysis; 590 peritoneal dialysis; 903 non-dialysis). Participants were more likely male (61.0%) with a median age of 61.5 years (IQR 50.0–72.7). Within 30 days of surgery, 272 people (8.0%) had an AMI or died. The probability was lowest following ophthalmologic surgery at 1.9% (95%CI: 0.5, 7.3) and kidney transplantation at 2.1% (95%CI: 1.3, 3.2). Several types of surgery were associated with greater than one in ten risk of AMI or death, including retroperitoneal (10.0% [95%CI: 2.5, 32.4]), intra-abdominal (11.7% [8.7, 15.5]), skin and soft tissue (12.1% [7.4, 19.1]), musculoskeletal (MSK) (12.3% [9.9, 15.5]), vascular (12.6% [10.2, 15.4]), anorectal (14.7% [6.3, 30.8]), and neurosurgical procedures (38.1% [20.3, 59.8]). Urgent or emergent procedures had the highest risk, with 12.1% experiencing AMI or death (95%CI: 10.7, 13.6) compared with 2.6% (1.9, 3.5) following elective surgery.

**Conclusions:**

After major non-cardiac surgery, the risk of death or AMI for people with kidney failure varies significantly based on surgery type. This study informs our understanding of surgery type and risk for people with kidney failure. Future research should focus on identifying high risk patients and strategies to reduce these risks.

**Supplementary Information:**

The online version contains supplementary material available at 10.1186/s12882-021-02577-7.

## Introduction

In high-income countries, 11% of adults undergo a surgical procedure annually [1, 2]. Though most of these procedures are uncomplicated, they can be associated with and contribute to significant morbidity and mortality, with an estimated 1 in 13 of all deaths globally occurring postoperatively [1]. Mechanisms of death in the perioperative period include postoperative infection, surgical complications, venous thromboembolism, bleeding, and death from cardiovascular (CV) causes [3, 4]. In high-income countries, postoperative CV events contribute to morbidity and increased cost associated with further intervention and hospitalization; further, death from CV causes is responsible for at least a third of perioperative deaths after non-cardiac surgery [5, 6]. Postoperative complications occur more frequently in older people and those with known CV disease or risk factors for CV disease [6, 7].

Kidney failure is defined by a sustained estimated glomerular filtration rate (eGFR) of < 15 mL/min/1.73m^2^ or receipt of chronic kidney replacement, and affects approximately 0.2% of North American adults [8, 9]. People with kidney failure often have numerous comorbidities with a high risk of CV disease, and account for a disproportionate amount of health care spending [8, 10]. Further, adults with kidney failure have an incidence of major surgery up to 16 times higher than that of people with normal eGFR [11]. Evidence related to perioperative outcomes for people with kidney failure primarily focuses on a single procedure or surgical category. These studies consistently suggest that people with kidney failure are at increased risk of adverse perioperative outcomes compared to those without kidney failure [12]. This extends to studies in general surgery [13–16], vascular surgery [17, 18], cardiac surgery [19–21], and orthopaedic surgery [22]. However, there are gaps in our understanding of outcomes in people with kidney failure. Many large perioperative databases, such as the American College of Surgeons National Surgical Quality Improvement Program (ACS NSQIP) [23], define dialysis patients as those receiving dialysis in the 2 weeks prior to surgery, which may misclassify and include people with acute kidney injury (AKI) requiring temporary dialysis. People with kidney failure who are not treated with kidney replacement are also not captured (i.e. those with eGFR < 15 mL/min/1.73m^2^).

We aimed to examine major non-cardiac surgery for people with kidney failure, both with and without dialysis, in a population-based cohort from Alberta, Canada. Our primary objective was to estimate the proportion of people with kidney failure that experienced the composite outcome of acute myocardial infarction (AMI) or death within 30 days of major non-cardiac surgery. Our secondary objectives were to examine the risk of the individual outcomes of death, AMI, and CV death associated with type of surgery.

## Methods

### Study design and setting

We assembled a retrospective, population-based cohort from the Alberta Kidney Disease Network (AKDN) database. This database maintains person-level linked administrative health data that includes ambulatory and inpatient datasets, physician claims, vital statistics, laboratory data, and renal program repositories with dialysis related records for the province of Alberta, Canada [24]. The population of Alberta is approximately 4.4 million people, with all residents eligible for public health insurance coverage. Over 99% have their health data captured in these administrative health databases [24, 25].

Our cohort included all adults (18 years or older) with eGFR less than 15 mL/min/1.73m^2^ or on chronic dialysis that had a major non-cardiac surgery in Alberta, Canada from April 1, 2005 to February 28, 2017, with outcome surveillance extending to March 31, 2017. All methods were conducted with a prespecified protocol, and followed the Strengthening the Reporting of Observational Studies in Epidemiology (STROBE) recommendations along with the Reporting of studies Conducted using Observational Routinely-collected health Data (RECORD) extension in the conduct and reporting of this cohort study (Supplementary Table 1) [26, 27]. We followed the ethical guidelines and regulations specified by the Conjoint Health Research Ethics Board at the University of Calgary and the Health Research Ethics Board at the University of Alberta, who both provided ethics approval and waived the need for informed consent.

### Participants and exposure

We defined our kidney disease cohort as either being in receipt of chronic dialysis or having non-dialysis dependent kidney failure with eGFR less than 15 mL/min/1.73m^2^. Chronic dialysis was defined as receiving hemodialysis or peritoneal dialysis for at least 90 days as an outpatient immediately before the index hospitalization for their surgical procedure. Non-dialysis kidney failure was defined as eGFR less than 15 mL/min/1.73m^2^, based on two consecutive outpatient measures of serum creatinine at least 90 days apart, with the most recent being within the year prior to the index surgical procedure. GFR was estimated using the CKD Epidemiology Collaboration (CKD-EPI) equation [28]. Though incident kidney transplant procedures were included in transplant-naïve patients with kidney failure (see below), people with prior kidney transplants (even if on chronic dialysis before the index procedure) were excluded. Kidney transplant patients are included in current kidney failure definitions [29], however types of surgery for this patient group are very different than dialysis patients and those not in receipt of kidney replacement therapy and so prevalent kidney transplant recipients were excluded.

Surgical procedures were defined based on the Canadian Classification of Health Interventions (CCI) coding [30]. These procedures were our exposure of interest and were categorized into 14 surgical groups as previously reported, into musculoskeletal (MSK), intra-abdominal, lower urologic/gynecologic, head and neck, vascular, skin and soft tissue, breast, neurosurgery, retroperitoneal, thoracic, anorectal, ophthalmologic, kidney transplant, and dialysis access surgery (arteriovenous fistula creation and peritoneal dialysis catheter insertion) [31–33]. The codes for surgical categories are included in Supplementary Table 2. Only therapeutic and not diagnostic procedures were included, and procedures that are typically performed as an outpatient (such as colonoscopy, bronchoscopy) were excluded as these are not surgeries and this exclusion is consistent with the literature. Major surgery was defined per previous literature as having a surgical procedure code that occurred during a hospitalization of at least one night in duration, or death on the same day as the surgical procedure [5, 6, 31, 34].

Only the first surgical procedure was included for each patient, to maintain independence of observations in our statistical analysis. Additionally, patients that left Alberta within the 30 days after the procedure were excluded.

### Variables: outcomes and covariates

Our primary outcome was a composite of all-cause mortality or AMI within 30 days of major non-cardiac surgery. Our prespecified secondary outcomes included each of: death within 30 days, AMI within 30 days (during index admission and for readmissions), and death from CV causes. As an exploratory analysis, we examined the risk of our primary outcome stratified into elective major surgery and urgent or emergent major surgery. Validated administrative data algorithms were used to define each of these outcomes, and are found in Supplementary Table 3.

We examined demographics, comorbidities, laboratory investigations, and surgical characteristics stratified by surgery type. Age as a continuous variable and sex (female and male) were obtained from the registry file. Kidney failure type was categorized as hemodialysis, peritoneal dialysis, or non-dialysis, and dialysis vintage in years was obtained from provincial renal program databases. Surgical urgency was determined using codes assigned to the hospital admission (planned procedures with pre-arranged admissions were categorized as elective). We also ascertained whether the cohort participant was admitted to hospital in the year prior to the procedure. A social deprivation index based on the 2011 and 2016 Canadian Census was used as a postal-code level correlate for socio-economic status [35]. Comorbidities were defined using validated algorithms based on International Statistical Classification of Diseases and Related Health Problems Ninth and Tenth Revision (ICD-9-CM and ICD-10-CA) codes [36] with an unrestricted lookback period, and are found in Supplementary Table 4. The most recent outpatient serum sodium, albumin, and hemoglobin in the year prior to the procedure were obtained from our linked laboratory databases if available.

### Statistical analyses

Baseline characteristics were summarized for the overall cohort and the surgical categories with counts and percentages for categorical or dichotomous variables, and medians and interquartile ranges (IQR) for continuous variables. All statistical analyses were completed using STATA software version 16.0 (StataCorp), with a two-sided statistical significance of *p* < 0.05 for all tests [37]. Our exposure was a 14-level categorical variable of surgical procedure type, and the count for each outcome within exposure strata was examined before statistical modelling. As there were too few events within many exposure categories for statistical modelling of our primary or secondary outcomes, we did not adjust our estimates in multivariable regression models, as was initially planned for our primary outcome. We instead used univariable logistic regression models to estimate the odds ratios (ORs) and 95% confidence intervals (CIs) for our primary outcome (AMI or death) and one of our secondary outcomes (death), which were then converted to proportions using postestimation commands in STATA.

## Results

### Cohort participants

**Fig. 1:**
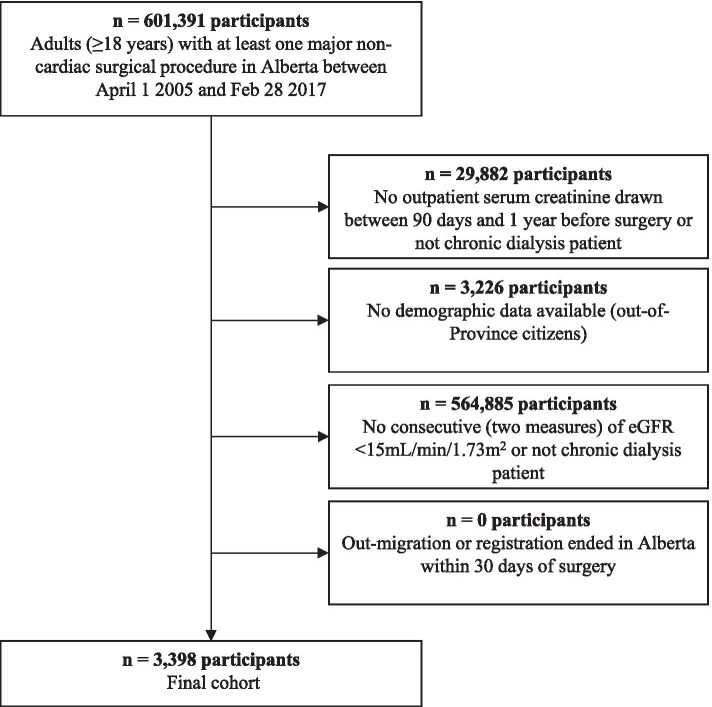
Cohort flow diagram. The number of patients that were included and excluded at each step of cohort formation are identified

Our initial cohort identified 601,391 adults that had at least one major non-cardiac surgery in Alberta between April 12,005 and February 282,017 (Fig. 1). Overall 597,993 people were excluded for not meeting our kidney failure criteria (94.5%), not having outpatient kidney function measured (5.0%), and not having demographics registered (0.5%). The final cohort included 3398 people with kidney failure who had undergone a major non-cardiac surgical procedure.

We summarize the baseline characteristics of the cohort in Table 1, and the characteristics by surgery category subgroup in Supplementary Table 5. The majority of the cohort were male (61.0%) with a median age of 61.5 years (IQR 50.0–72.7). Most were hemodialysis recipients (56.1%) with a median dialysis vintage of 2.3 years (IQR 1.2–4.1). The most common surgery type was kidney transplantation (*n* = 923 procedures, 27.2%), followed by MSK and vascular procedures (both *n* = 627 procedures, 18.5%). There were more urgent or emergent major surgeries (56.9%) compared with elective major surgeries. The most common comorbidities included hypertension (94.2%) and diabetes (52.2%).

**Table 1:** Cohort Baseline Characteristics and Overall Outcome Frequency

	Total (***n*** = 3398)	
Characteristic	No.	%
Female Sex	1326	39
Age (years; IQR)	61.5 (50.0–72.7)	
Kidney Failure Type		
Non-dialysis	903	26.6
Hemodialysis	1905	56.1
Peritoneal dialysis	590	17.4
Dialysis vintage (*n* = 2495; years, IQR)	2.3 (1.2–4.1)	
Surgery Type		
Kidney Transplant	923	27.2
Musculoskeletal	627	18.5
Vascular	627	18.5
Intra-abdominal	351	10.3
Dialysis Access	262	7.7
Head and Neck	192	5.7
Skin and Soft Tissue	124	3.6
Ophthalmology	105	3.1
Lower Urologic and Gynecologic	61	1.8
Anorectal	34	1.0
Thoracic	29	0.9
Breast	22	0.6
Neurosurgery	21	0.6
Retroperitoneal	20	0.6
Procedure Urgency		
Elective	1463	43.1
Urgent/Emergent	1935	56.9
Hospitalized in prior year	1639	48.2
Comorbidities		
Cancer	795	23.4
Cerebrovascular Disease	838	24.7
Congestive Heart Failure	1442	42.4
COPD	1652	48.6
Dementia	239	7.0
Diabetes	1774	52.2
Hypertension	3200	94.2
Liver Disease	377	11.1
Myocardial Infarction	1025	30.2
Obesity	498	14.7
Paraplegia	152	4.5
Peptic Ulcer Disease	614	18.1
Peripheral Vascular Disease	1508	44.4
Rheumatologic Disease	336	9.9
Social Deprivation Index		
Least deprived	525	15.5
2	414	12.2
3	544	16.0
4	756	22.3
Most deprived	927	27.3
Not defined	231	6.8
Serum sodium (mmol/L; IQR)	137 (134–139)	
Missing	615	18.1
Serum albumin (g/L)	36 (32–39)	
Missing	654	19.2
Serum hemoglobin (g/L)	109 (98–118)	
Missing	3	0.1
Outcomes		
AMI or Death in 30 days	272	8.0
Death in 30 days	181	5.3
AMI in 30 days	113	3.3
CV Death in 30 days	32	0.9

*AMI* acute myocardial infarction; *COPD* chronic obstructive pulmonary disease; *CV* cardiovascular; *g/L* grams per litre; *IQR* interquartile range; millimoles per litre; *No* number

### Primary outcome: composite of AMI or death within 30 days of major non-cardiac surgery

**Fig. 2:**
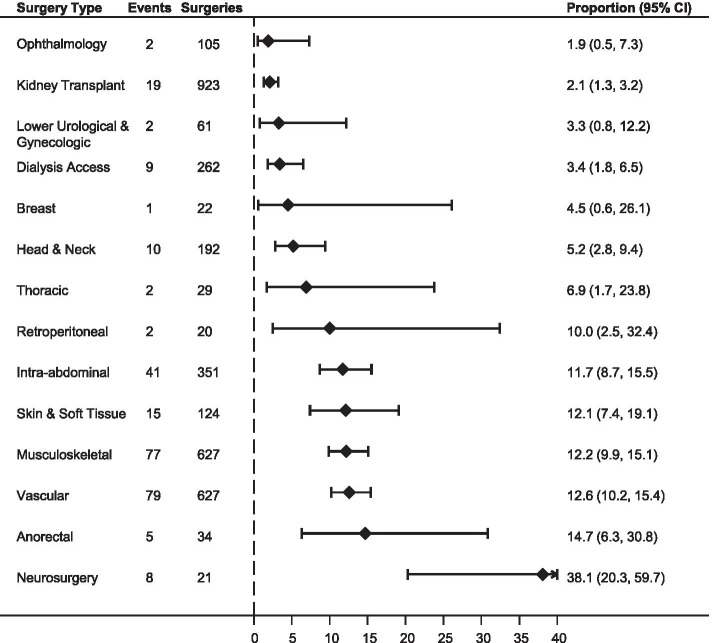
Proportion with Acute Myocardial Infarction or Death within 30 days of Major Non-Cardiac Surgery. The unadjusted proportions of acute myocardial infarction (AMI) or death within 30 days of major non-cardiac surgery are presented with their 95% confidence intervals, stratified by surgery type

In the 30 days after major non-cardiac surgery, 272 people died or had an AMI (8.0% of our cohort; 181 deaths of which 22 were with AMI, 91 non-fatal AMI). Most of these events (*n* = 234, 86.0%) occurred in patients who had an urgent procedure. The proportion of people experiencing death or AMI was lowest following ophthalmologic surgery at 1.9% (95%CI: 0.5, 7.3) and kidney transplantation at 2.1% (95% CI: 1.3, 3.2). There were six surgery types with proportions above 10%, including retroperitoneal (10.0% [95%CI: 2.5, 32.4]), intra-abdominal (11.7% [8.7, 15.5]), skin and soft tissue (12.1% [7.4, 19.1]), musculoskeletal (MSK) (12.3% [9.9, 15.5]), vascular (12.6% [10.2, 15.4]), anorectal (14.7% [6.3, 30.8]), and neurosurgical procedures (38.1% [20.3, 59.8]) (Fig. 2, Table 2). Presentation of model fit statistics along with estimates as odds ratios is found in Supplementary Table 6. When we stratified the estimates of our primary outcome by surgical urgency, the proportion of AMI or death was highest overall and for most surgery subtypes if performed on an urgent or emergent basis (Table 2). Urgent or emergent surgery was associated with AMI or death in 12.1% (95%CI: 10.7, 13.6) of surgeries compared with 2.6% (1.9, 3.5) following elective major surgery.

**Table 2:** Proportions of AMI or Death for people with kidney surgery based on type of surgery, stratified into elective surgery and urgent/emergent surgery subgroups

Surgical Category	Number of Surgeries	Number of Elective Surgeries	Number of Urgent or Emergent Surgeries	Number of AMI + Death Events Overall	Proportion (%) of AMI and Death within 30 days (95%CI)	
					Elective Surgery Subgroup	Urgent or Emergent Surgery Subgroup
Ophthalmology	105	69	36	2	1.0 (0.2, 4.1)	3.6 (0.9, 13.3)
Kidney Transplant	923	595	328	19	1.1 (0.6, 1.8)	3.8 (2.4, 6.)
Lower Urologic and Gynecologic	61	52	9	2	3.3 (0.8, 12.2)	1.6 (0.2, 10.7)
Dialysis Access	262	114	148	9	1.4 (0.7, 2.9)	5.0 (2.6, 9.3)
Breast	22	19	3	1	4.5 (0.6, 26.1)	N/A
Head and Neck	192	146	46	10	3.4 (1.7, 6.3)	11.1 (6.0, 19.6)
Thoracic	29	12	17	2	2.9 (0.7, 11.6)	9.7 (2.5, 31.5)
Retroperitoneal	20	10	10	2	4.8 (1.1, 18.4)	15.2 (3.9, 44.3)
Intra-abdominal	351	94	257	41	4.4 (2.8, 6.9)	14.3 (10.7, 18.9)
Skin and Soft Tissue	124	21	103	15	4.2 (2.3, 7.7)	13.7 (8.4, 21.5)
Musculoskeletal	627	158	469	77	4.6 (3.1, 6.8)	14.9 (12.0, 18.2)
Vascular	627	152	475	79	4.7 (3.2, 6.9)	15.1 (12.3, 18.5)
Anorectal	34	16	18	5	7.1 (2.7, 17.2)	21.5 (9.4, 42.0)
Neurosurgery	21	5	16	8	18.1 (7.8, 36.6)	44.3 (24.3, 66.4)
**Overall**	**3398**	**1463**	**1935**	**272**	**2.6 (1.9, 3.5)**	**12.1 (10.7, 13.6)**

*AMI* Acute Myocardial Infarction; *CI* confidence interval

### Secondary outcomes: death within 30 days; AMI within 30 days; CV death within 30 days

There were 181 people (5.3%) that died within 30 days of major non-cardiac surgery. Kidney transplantation was associated with lowest proportion of postoperative death, at 0.2% (95%CI: 0.05, 0.9). In comparison, there was a statistically higher proportion of people that died after head and neck (2.6% [1.1, 6.1]), thoracic (6.9% [1.7, 23.8]), intra-abdominal (8.0% [5.6, 11.3]), vascular (8.3% [6.4, 10.7]), anorectal (8.8% [2.9, 24.0]), skin and soft tissue (8.9% [5.0, 15.3]), MSK (9.7% [7.6, 12.3]), and neurosurgical procedures (33.3% [16.8, 55.3]) (Table 3). The most common causes of death overall included atherosclerotic heart disease (16, 9%), kidney failure (13, 7%), and unspecified diabetes complications (11, 6%) (Supplementary Table 7).

**Table 3:** Proportions of Composite Outcome of AMI or Death, and Death alone, for people with kidney failure undergoing major non-cardiac surgery, by surgery type

Surgical Category	Number of People with Surgeries	Number of AMI + Death Events	Proportion (%) of AMI and Death within 30 days (95%CI)	Number of Death Events	Proportion (%) of Death within 30 days (95%CI)
Ophthalmology	105	2	1.9 (0.5, 7.3)	2	1.9 (0.5, 7.3)
Kidney Transplant	923	19	2.1 (1.3, 3.2)	2	0.2 (0.05, 0.9)
Lower Urologic and Gynecologic	61	2	3.3 (0.8, 12.2)	1	1.6 (0.2, 10.7)
Dialysis Access	262	9	3.4 (1.8, 6.5)	5	1.9 (0.8, 4.5)
Breast	22	1	4.5 (0.6, 26.1)	1	4.5 (0.6, 26.1)
Head and Neck	192	10	5.2 (2.8, 9.4)	5	2.6 (1.1, 6.1)
Thoracic	29	2	6.9 (1.7, 23.8)	2	6.9 (1.7, 23.8)
Retroperitoneal	20	2	10.0 (2.5, 32.4)	1	5.0 (0.7, 28.2)
Intra-abdominal	351	41	11.7 (8.7, 15.5)	28	8.0 (5.6, 11.3)
Skin and Soft Tissue	124	15	12.1 (7.4, 19.1)	11	8.9 (5.0, 15.3)
Musculoskeletal	627	77	12.2 (9.9, 15.1)	61	9.7 (7.6, 12.3)
Vascular	627	79	12.6 (10.2, 15.4)	52	8.3 (6.4, 10.7)
Anorectal	34	5	14.7 (6.3, 30.8)	3	8.8 (2.9, 24.0)
Neurosurgery	21	8	38.1 (20.3, 59.7)	7	33.3 (16.8, 55.3)
Number included in analysis	–	–	3398	–	3398
Bayesian Information Criterion (BIC)	–	–	1871.9	–	1374.1
McFadden’s Adjusted Pseudo-R2	–	–	0.072	–	0.089

*AMI* Acute Myocardial Infarction; *CI* confidence interval

There were 113 people who experienced AMI and 32 people who died of CV causes within 30 days of major non-cardiac surgery. Given the low number of events for many surgical types, statistical comparisons among procedure types were not conducted as per our prespecified statistical plans.

## Discussion

In this population-based cohort study we examined postoperative outcomes of major non-cardiac surgery for people with kidney failure from Alberta, Canada, and found that 8.0% died or had an AMI within 30 days of surgery. Kidney transplantation was the most frequent procedure and was associated with one of the lowest estimated probabilities of this outcome, with higher proportions following head and neck, thoracic, vascular, skin and soft tissue, intra-abdominal, musculoskeletal, retroperitoneal, anorectal, and neurosurgical procedures. The risk was highest for urgent or emergent major surgery.

Our primary objective was to determine the magnitude of risk that people with kidney failure face, both those with and without receipt of dialysis, when they undergo major non-cardiac surgery. We found that an estimated one in twelve people with kidney failure died or had an AMI within 30 days of their index procedure. Most of these events were deaths (*n* = 181, 5.3%), which is consistent with previous literature that has examined the perioperative experience of this patient population. A recent meta-analysis compared postoperative mortality between chronic dialysis patients and those with normal kidney function [12]. They found that the pooled adjusted odds of death within 30 days was more than five-fold higher than hose with normal kidney function. Further, they found that the absolute risk of death differed for dialysis patients based on surgical type. Urological and gynecological procedures had an absolute mortality risk of 0.6% (95%CI: 0.0–1.8), orthopaedic procedures 3.4% (95%CI: 1.8–6.4), general surgical procedures 3.8% (95%CI: 1.0–9.1), vascular procedures 7.8% (95%CI: 4.0–14.3), and cardiac surgical procedures 8.7% (95%CI: 4.8–11.1). Our own examination of categorical surgery subtypes revealed important differences in associated risk, and are consistent with their results. Notably, we found that kidney transplantation was associated with one of the lowest probabilities of death or AMI within 30 days of surgery. Compared with kidney transplant, head and neck, vascular, skin and soft tissue, intra-abdominal, musculoskeletal, retroperitoneal, anorectal, and neurosurgical procedures all had higher proportions of death and AMI. In a cohort study derived from the National Inpatient Sample in the United States, Smilowitz et al. compared the occurrence of in-hospital death and CV events between types of solid organ transplants and non-transplant surgeries [38]. They found that kidney transplantation was associated with a lower risk of perioperative death than other non-transplant related procedures in the general population [38]. This is not surprising, as kidney transplant recipients are highly selected with extensive pre-transplant cardiac risk evaluation and screening prior to surgery [39]. Further, these findings likely translate to other elective surgeries performed in our cohort, which are more likely to be performed in people that have received some form of preoperative cardiac risk stratification. Overall, with the results of our study and other literature, it has been consistently demonstrated that surgery type is an important risk factor for postoperative outcomes after major surgery. Additionally, some of the more common surgery types have probabilities of death or AMI which may be surprising to people with kidney failure and their healthcare providers. Almost one in eight people undergoing MSK, vascular, skin and soft tissue, or intra-abdominal procedures that require admission to hospital die or have an AMI. Many risk stratification tools incorporate some component of surgery type, though often as a dichotomized variable based on risk. Our results suggest that the risk associated with this variable is more heterogeneous, which has implications for how it should be incorporated into future risk prediction models in this population.

In our study, we identified 113 post-operative AMI events (3.3%). Smilowitz et al. found that kidney transplant procedures had lower adjusted odds of death and stroke, but two-fold higher odds of AMI when compared to other procedures (again, not limited to those with kidney failure). In absolute risk, 0.5% of kidney transplant recipients died in hospital in their study, and 1.2% had an AMI during their index transplant admission. In another study from the same group, perioperative AMI occurred in approximately 2.7% of people with kidney failure after major non-cardiac surgery [32]. These estimates of cardiac event risk are similar to our own. Both of our studies used the same algorithms to identify ST-segment elevation MIs (STEMIs) and Non-STEMIs. These codes should not identify less severe presentations which may be asymptomatic, highlighting the difficulty in ascertaining postoperative AMIs from administrative data sources. With the degree of baseline cardiac enzyme elevation in the kidney failure population, diagnosis of asymptomatic perioperative AMIs is challenging [40]. Routine monitoring of cardiac biomarkers in higher risk patients postoperatively has only been recently recognized as important to identify otherwise asymptomatic myocardial injury, and recommended by our national perioperative risk stratification guidelines [5]. Importantly, this monitoring practice was not in place during the period of this cohort study and therefore we likely underestimated perioperative CV event occurrence.

Our findings are strengthened in several ways. We used a population-based cohort to examine our research question, with near universal health care coverage. We used a rigorous definition of kidney failure to include people both in receipt and not in receipt of dialysis, to ensure that our cohort definition and the primary outcome of interest were resilient to temporal changes in dialysis practices. Our primary outcome was specifically chosen as a composite to reduce the effects of competing risks, and to be analogous to common perioperative risk composites, which generally combine death and major CV events together [23, 34]. However, our results must be interpreted with acknowledgement of limitations intrinsic to use of administrative health data. Though we used validated algorithms to define comorbidities and our outcomes, these algorithms may not be sensitive to identifying all cases, and misclassification of nonfatal events may lead to underestimation of outcome occurrence. Further, as mentioned, less severe presentations of postoperative CV events (i.e. myocardial injury after non-cardiac surgery [MINS]) will not be identified with existing ICD code algorithms. Given limitations in data availability, we were not able to examine unmeasured variables of interest such as pre-operative surgical disease severity, degree and type of risk stratification, preoperative lifestyle variables like smoking, or specific causes of postoperative death. Generally, elective surgeries are more likely to have been preceded by preoperative risk stratification, and perioperative risk will be impacted. As our surgical urgency variable was dependent on the patient not having a planned (or prebooked) admission, some urgent procedures such as colorectal surgery resection may have been misclassified as elective. Further, though we did include important and population-specific variables such as age, procedure urgency, kidney failure type, serum sodium and albumin, and several comorbidities, the limited number of outcomes made it statistically inappropriate to adjust estimates for these variables. It is also possible that following an unsuccessful major surgery, some people with kidney failure may withdraw from dialysis. This may lead to a greater proportion of death postoperatively, though this is difficult to ascertain from our existing data sources. We did not compare the kidney failure population to people without kidney failure, as our objective was to examine differences in postoperative outcomes by surgery type in people with kidney failure. Future work should examine whether level of kidney function modifies the differences in risk that we found. Finally, as we examined the risk associated with procedures that were performed in the kidney failure population, our study likely underestimates the risk for all people with kidney failure that have a surgical indication since many high-risk individuals may be deemed unfit or too comorbid for surgery.

In conclusion, in our population-based cohort study we determined the surgery-type specific probability of death and CV events after major non-cardiac surgery for people with kidney failure and found that overall surgical risk varies significantly based on surgery type. Most of these postoperative outcomes were experienced by those with urgent major surgery, with relatively few outcomes occurring after elective major surgery. Our results are generalizable to other populations where universal health care insurance is provided to people with kidney failure, who have similar surgical access and preoperative risk assessment. Future research is needed to understand how surgery type interacts with patient factors to impact risk of postoperative outcomes, including kidney disease severity, and procedure level variables such as whether the procedure was a repeat surgery. This work is necessary to inform strategies to reduce these risks.

## Supplementary Information

**Additional file 1:.**

## Data Availability

This study is based in part on data provided by Alberta Health and Alberta Health Services, and is not publicly available for sharing. The interpretation and conclusions contained herein are those of the researchers and do not necessarily represent the views of the Government of Alberta or Alberta Health Services. Neither the Government of Alberta, Alberta Health or Alberta Health Services express any opinion in relation to this study. We are not able to make our dataset available to other researchers due to our contractual arrangements with the provincial health ministry (Alberta Health), who is the data custodian. Researchers may make requests to obtain a similar dataset at https://www.alberta.ca/health-research.aspx.
