# Supplementary material for: Mortality and cardiovascular events in adults with kidney failure after major non-cardiac surgery: a population-based cohort study
Source: BMC Nephrol. 2021 Nov 4;22:365. doi: 10.1186/s12882-021-02577-7 (PMC8569960; doi:10.1186/s12882-021-02577-7)
Supplement: Supplementary file 1 — Additional file 1:. [file 12882_2021_2577_MOESM1_ESM.docx]

Mortality and cardiovascular events in adults with kidney failure after major non-cardiac surgery: A Population-Based Cohort Study

Harrison TG et al.

**Supplementary Material**

**Supplementary Table 1.** Strengthening the Reporting of Observational Studies in Epidemiology (STROBE) and Reporting of studies Conducted using Observational Routinely-collected health Data (RECORD) extension checklist.

**Supplementary Table 2.** Surgical Categories by Canadian Classification of Health Intervention (CCI) codes, with section and group +/- intervention level of ICD-10 codes

**Supplementary Table 3.** Algorithms of ICD-9 and 10 codes used to define primary and secondary outcomes

**Supplementary Table 4.** Variables that were considered as potential confounders of the association of interest, along with source of data and ICD-9/10 algorithms

**Supplementary Table 5.** Cohort Baseline Characteristics, stratified by procedure type

**Supplementary Table 6.** Unadjusted Odds of AMI and Death for people with kidney failure undergoing major non-cardiac surgery, stratified by surgical procedure type

**Supplementary Table 7.** Top Causes of Perioperative Death for people with Kidney Failure

**Supplementary Table 1.** Strengthening the Reporting of Observational Studies in Epidemiology (STROBE) and Reporting of studies Conducted using Observational Routinely-collected health Data (RECORD) extension checklist.

|  | **Item No.** | **STROBE items** | **Location in manuscript where items are reported** | **RECORD items** | **Location in manuscript where items are reported** |
| --- | --- | --- | --- | --- | --- |
| **Title and abstract** | | | | | |
|  | 1 | (a) Indicate the study’s design with a commonly used term in the title or the abstract (b) Provide in the abstract an informative and balanced summary of what was done and what was found | 1, 5-6 | RECORD 1.1: The type of data used should be specified in the title or abstract. When possible, the name of the databases used should be included.  RECORD 1.2: If applicable, the geographic region and timeframe within which the study took place should be reported in the title or abstract.  RECORD 1.3: If linkage between databases was conducted for the study, this should be clearly stated in the title or abstract. | 5  5  5 |
| **Introduction** | | | | | |
| Background rationale | 2 | Explain the scientific background and rationale for the investigation being reported | 7,8 |  |  |
| Objectives | 3 | State specific objectives, including any prespecified hypotheses | 8 |  |  |
| **Methods** | | | | | |
| Study Design | 4 | Present key elements of study design early in the paper | 9 |  |  |
| Setting | 5 | Describe the setting, locations, and relevant dates, including periods of recruitment, exposure, follow-up, and data collection | 9 |  |  |
| Participants | 6 | *(a) Cohort study* - Give the eligibility criteria, and the sources and methods of selection of participants. Describe methods of follow-up  *Case-control study* - Give the eligibility criteria, and the sources and methods of case ascertainment and control selection. Give the rationale for the choice of cases and controls  *Cross-sectional study* - Give the eligibility criteria, and the sources and methods of selection of participants  *(b) Cohort study* - For matched studies, give matching criteria and number of exposed and unexposed  *Case-control study* - For matched studies, give matching criteria and the number of controls per case | 9, 10  N/A | RECORD 6.1: The methods of study population selection (such as codes or algorithms used to identify subjects) should be listed in detail. If this is not possible, an explanation should be provided.  RECORD 6.2: Any validation studies of the codes or algorithms used to select the population should be referenced. If validation was conducted for this study and not published elsewhere, detailed methods and results should be provided.  RECORD 6.3: If the study involved linkage of databases, consider use of a flow diagram or other graphical display to demonstrate the data linkage process, including the number of individuals with linked data at each stage. | 9, 10, Supplementary tables 2-4  Supplementary table 3  N/A |
| Variables | 7 | Clearly define all outcomes, exposures, predictors, potential confounders, and effect modifiers. Give diagnostic criteria, if applicable. | 9-11 | RECORD 7.1: A complete list of codes and algorithms used to classify exposures, outcomes, confounders, and effect modifiers should be provided. If these cannot be reported, an explanation should be provided. | Supplementary Tables 2-4 |
| Data sources/ measurement | 8 | For each variable of interest, give sources of data and details of methods of assessment (measurement).  Describe comparability of assessment methods if there is more than one group | 9-11 |  |  |
| Bias | 9 | Describe any efforts to address potential sources of bias | 12 |  |  |
| Study size | 10 | Explain how the study size was arrived at | N/A (population-based cohort) |  |  |
| Quantitative variables | 11 | Explain how quantitative variables were handled in the analyses. If applicable, describe which groupings were chosen, and why | 9-12 |  |  |
| Statistical methods | 12 | (a) Describe all statistical methods, including those used to control for confounding  (b) Describe any methods used to examine subgroups and interactions  (c) Explain how missing data were addressed  (d) *Cohort study* - If applicable, explain how loss to follow-up was addressed  *Case-control study* - If applicable, explain how matching of cases and controls was addressed  *Cross-sectional study* - If applicable, describe analytical methods taking account of sampling strategy  (e) Describe any sensitivity analyses | 11-12  11  11-12  N/A |  |  |
| Data access and cleaning methods |  | .. |  | RECORD 12.1: Authors should describe the extent to which the investigators had access to the database population used to create the study population.  RECORD 12.2: Authors should provide information on the data cleaning methods used in the study. | 9  N/A (see reference Hemmelgarn et al. ^1^) |
| Linkage |  | .. |  | RECORD 12.3: State whether the study included person-level, institutional-level, or other data linkage across two or more databases. The methods of linkage and methods of linkage quality evaluation should be provided. | 9 |
| **Results** | | | | | |
| Participants | 13 | (a) Report the numbers of individuals at each stage of the study (*e.g.*, numbers potentially eligible, examined for eligibility, confirmed eligible, included in the study, completing follow-up, and analysed)  (b) Give reasons for non-participation at each stage.  (c) Consider use of a flow diagram | 13, Fig 1 | RECORD 13.1: Describe in detail the selection of the persons included in the study (*i.e.,* study population selection) including filtering based on data quality, data availability and linkage. The selection of included persons can be described in the text and/or by means of the study flow diagram. | Fig 1 |
| Descriptive data | 14 | (a) Give characteristics of study participants (*e.g.*, demographic, clinical, social) and information on exposures and potential confounders  (b) Indicate the number of participants with missing data for each variable of interest  (c) *Cohort study* - summarise follow-up time (*e.g.*, average and total amount) | 13, Table 1, Supp Table 5  13-14, Table 1, Supp Table 5  N/A as 30 day outcome surveillance period |  |  |
| Outcome data | 15 | *Cohort study* - Report numbers of outcome events or summary measures over time  *Case-control study* - Report numbers in each exposure category, or summary measures of exposure  *Cross-sectional study* - Report numbers of outcome events or summary measures | 13-14 |  |  |
| Main results | 16 | (a) Give unadjusted estimates and, if applicable, confounder-adjusted estimates and their precision (e.g., 95% confidence interval). Make clear which confounders were adjusted for and why they were included  (b) Report category boundaries when continuous variables were categorized  (c) If relevant, consider translating estimates of relative risk into absolute risk for a meaningful time period | 13-14, Table 2, Table 3, Figure 2 |  |  |
| Other analyses | 17 | Report other analyses done—e.g., analyses of subgroups and interactions, and sensitivity analyses | 13, 14, Supp Table 6 |  |  |
| **Discussion** | | | | | |
| Key results | 18 | Summarise key results with reference to study objectives | 14-15 |  |  |
| Limitations | 19 | Discuss limitations of the study, taking into account sources of potential bias or imprecision. Discuss both direction and magnitude of any potential bias | 17 | RECORD 19.1: Discuss the implications of using data that were not created or collected to answer the specific research question(s). Include discussion of misclassification bias, unmeasured confounding, missing data, and changing eligibility over time, as they pertain to the study being reported. | 17 |
| Interpretation | 20 | Give a cautious overall interpretation of results considering objectives, limitations, multiplicity of analyses, results from similar studies, and other relevant evidence | 17 |  |  |
| Generalisability | 21 | Discuss the generalisability (external validity) of the study results | 17 |  |  |
| **Other Information** | | | | | |
| Funding | 22 | Give the source of funding and the role of the funders for the present study and, if applicable, for the original study on which the present article is based | 3 |  |  |
| Accessibility of protocol, raw data, and programming code |  | .. |  | RECORD 22.1: Authors should provide information on how to access any supplemental information such as the study protocol, raw data, or programming code. | 3 |

*Reference: Benchimol EI, Smeeth L, Guttmann A, Harron K, Moher D, Petersen I, Sørensen HT, von Elm E, Langan SM, the RECORD Working Committee. The REporting of studies Conducted using Observational Routinely-collected health Data (RECORD) Statement. *PLoS Medicine* 2015; in press.

*Checklist is protected under Creative Commons Attribution ([CC BY](http://creativecommons.org/licenses/by/4.0/)) license.

**Supplementary Table 2.** Surgical Categories by Canadian Classification of Health Intervention (CCI) codes, with section and group +/- intervention level of ICD-10 codes from Hospitalization data

| **Surgery Type** | **Section, Group, Intervention Components of ICD-10 Codes based on Canadian Classification of Interventions (CCI)** |
| --- | --- |
| Anorectal | 1NQ56DA; 1NQ56LA; 1NQ59DAAD; 1NQ59DAAG; 1NQ59DAGX; 1NQ59HAX7; 1NQ59LAAD; 1NQ59LAAG; 1NQ59LAGX; 1NQ72DA; 1NQ72LA; 1NQ72PB; 1NQ74DW; 1NQ74ED; 1NQ74EJ; 1NQ74PC; 1NQ74PD; 1NQ74PE; 1NQ74SS; 1NQ74TV; 1NQ74VT; 1NQ80; 1NQ84; 1NQ86; 1NQ87CA; 1NQ87DA; 1NQ87DF; 1NQ87DX; 1NQ87LA; 1NQ87PB; 1NQ87PF; 1NQ87RD; 1NQ87TF; 1NQ89; 1NQ90; 1NT53LADV; 1NT53LAPM; 1NT56LA; 1NT72; 1NT80; 1NT84; 1NT86; 1NT87 |
| Breast | 1YK50; 1YK58; 1YK80; 1YK83; 1YK84; 1YK87; 1YK89; 1YK90; 1YL87; 1YL89; 1YM58; 1YM74; 1YM78; 1YM79; 1YM80; 1YM87; 1YM88; 1YM89; 1YM90; 1YM91; 1YM92 |
| Dialysis-related Access (Vascular access [fistula and graft] and Peritoneal Dialysis access) | 1OT53; 1KY76LA; 1KY76LASJ; 1KY76LAXXA; 1KY76LAXXL; 1KY76LAXXN; 1YY53LA-TS (buried PD catheter) |
| Head and Neck | 1DA56LA; 1DA58; 1DA59HAT9; 1DA59JAGX; 1DA80; 1DA82; 1DA83; 1DA84; 1DA87; 1DA89; 1DA91; 1DE56LA; 1DE59JAGX; 1DE80; 1DE84; 1DE86; 1DE87; 1DE91; 1DF53; 1DF58; 1DF71JA; 1DF72LA; 1DF80; 1DF85; 1DF87; 1DF89; 1DG; 1DJ53; 1DJ80; 1DK59LAGX; 1DK80; 1DK85; 1DK87; 1DK91; 1DE53; 1DL59LAGX; 1DL80; 1DL87; 1DL89; 1DL91; 1DM53; 1DN76QRQB; 1DP53; 1DR57; 1DR59LAKD; 1DR59QRAZ; 1DR59QRKD; 1DR59QRX7; 1DR72; 1DR80; 1DR89; 1DR91; 1DZ70; 1DZ94; 1EA56; 1EA58; 1EA72; 1EA73; 1EA74; 1EA80; 1EA87; 1EA92; 1EB73LA; 1EB74; 1EB80; 1EB87; 1EC; 1ED56; 1ED73; 1ED74; 1ED79; 1ED80; 1ED83; 1ED87; 1ED91; 1EE56; 1EE58; 1EE71; 1EE73; 1EE78; 1EE79; 1EE80; 1EE83; 1EE87; 1EE91; 1EF73; 1EF74; 1EF80; 1EG; 1EH; 1EJ87; 1EJ89; 1EL53; 1EL57LA; 1EL72DA; 1EL72LA; 1EL74; 1EL80; 1EL83; 1EM53; 1EM73LA; 1EM74; 1EM80; 1EM86; 1EM87; 1EN53; 1EN73LA; 1EN74; 1EN80; 1EN87; 1EN91; 1EP58; 1EP72; 1EP80; 1EP87; 1EQ56LA; 1EQ59; 1EQ70; 1EQ87; 1EQ94; 1ES58; 1ES80; 1ES87; 1ET56LA; 1ET57LA; 1ET59LAAD; 1ET59LAAG; 1ET59LAGX; 1ET72LA; 1ET73; 1ET80; 1ET82; 1ET86; 1ET87; 1ET89; 1EU87; 1EU89; 1EV87; 1EW80; 1EW86; 1EW87; 1EW91; 1EX59; 1EX80; 1EX87; 1EY87; 1EY91; 1FA53; 1FA56LA; 1FA84; 1FA87; 1FA91; 1FB53; 1FB80; 1FB86; 1FB87; 1FB91; 1FC56LA; 1FC80LAXXB; 1FC80LAXXE; 1FC87; 1FG56LA; 1FG58; 1FG59JAGX; 1FG72; 1FG80; 1FG87; 1FH56LA; 1FH59JAGX; 1FH78; 1FH80; 1FH87; 1FJ56LA; 1FJ59JAGX; 1FJ72; 1FJ74; 1FJ80; 1FJ87; 1FJ91; 1FK94; 1FL51; 1FL57; 1FL80; 1FL87; 1FL89; 1FM50; 1FM51; 1FM57; 1FM80; 1FM83; 1FM87; 1FM89; 1FM91; 1FN51; 1FN57; 1FN59; 1FN80; 1FN83; 1FN87; 1FN89; 1FQ56LA; 1FQ59HAAW; 1FQ78; 1FQ80; 1FQ87; 1FQ89; 1FR56LA; 1FR59JAGX; 1FR87; 1FR89; 1FU71; 1FU87; 1FU89; 1FU91; 1FV83; 1FV87; 1FV89; 1FX56LA; 1FX80; 1FX86; 1FX87; 1FX91; 1GA74; 1GA80; 1GA83; 1GA87; 1GA89; 1GB; 1GC50; 1GC59; 1GD53; 1GD74; 1GD83; 1GD87; 1GD89; 1GE50; 1GE56LA; 1GE80; 1GE87; 1GE89; 1GE91; 1GH71; 1GH84; 1GJ50LA; 1GJ50LANR; 1GJ53LAPM; 1GJ56LA; 1GJ77; 1GJ80; 1GJ82; 1GJ85; 1GJ86; 1GJ87; 1GK52LA; 1GK59LAGX; 1GK74LA; 1GK80; 1GK83; 1GK87; 1GK89; 1MB87LA; 1MC87; 1MC89; 1MC91; 1ML59; 1ML87 |
| Intra-abdominal | 1MG87; 1MG89; 1MJ87; 1MJ89; 1MJ91; 1MP50; 1MP51; 1MP59; 1MP76; 1MP80; 1MP87; 1NF80DAXXE; 1NF80DAXXN; 1NF80LA; 1NF80LAXXE; 1NF80LAXXN; 1NF82; 1NF84; 1NF86; 1NF87; 1NF89; 1NF90; 1NF91; 1NF92; 1NK53DATS; 1NK53LAQB; 1NK53LATS; 1NK56DA; 1NK56LA; 1NK58; 1NK74; 1NK76; 1NK77; 1NK80; 1NK82; 1NK84; 1NK85; 1NK87DA; 1NK87DN; 1NK87DP; 1NK87DX; 1NK87DY; 1NK87LA; 1NK87RE; 1NK87RF; 1NK87TF; 1NK87TG; 1NM56DA; 1NM56LA; 1NM58; 1NM74; 1NM76; 1NM77; 1NM80; 1NM82; 1NM87; 1NM89; 1NM91; 1NP58; 1NP72; 1NP73LA; 1NP85; 1NP86; 1NV89; 1OA53; 1OA58; 1OA59DAGX; 1OA59LAGX; 1OA74; 1OA85; 1OA87; 1OB59DAGX; 1OB59LAGX; 1OB74; 1OB83; 1OB85; 1OB87; 1OB89; 1OD57; 1OD76; 1OD80; 1OD86; 1OD89; 1OE57DAAG; 1OE57DAAM; 1OE57DAAS; 1OE57DAAZ; 1OE57DABD; 1OE57DAGX; 1OE57HAAG; 1OE57HAAM; 1OE57HAAS; 1OE57HAAZ; 1OE57HABD; 1OE57HAGX; 1OE57LAAG; 1OE57LAAM; 1OE57LAAS; 1OE57LAAZ; 1OE57LABD; 1OE57LAGX; 1OE59KQ; 1OE76; 1OE80; 1OE84; 1OE86; 1OE87; 1OE89; 1OJ53; 1OJ56; 1OJ76; 1OJ83; 1OJ85; 1OJ87; 1OJ89; 1OK58; 1OK85; 1OK87; 1OK89; 1OK91; 1OT56; 1OT58; 1OT70; 1OT72; 1OT80; 1OT87; 1OT91; 1OW12; 1OW80DA; 1OW80LA; 1OW87; 1OW89; 1OZ94LA |
| Kidney transplant | 1PC85 |
| Lower Urologic/Gynecologic | 1MH87; 1MH89; 1PQ53LAPZ; 1PQ56DA; 1PQ56LA; 1PQ56QY; 1PQ57LAAM; 1PQ57LAGX; 1PQ59FLAD; 1PQ59FLAG; 1PQ59FLAS; 1PQ59FLAZ; 1PQ59FLGX; 1PQ59LAAZ; 1PQ59LAGX; 1PQ72; 1PQ77; 1PQ78; 1PQ80; 1PQ82; 1PQ86; 1PQ87; 1PQ89; 1PV57LAGX; 1PV59LAGX; 1PV80; 1PZ94DA; 1PZ94HA; 1PZ94LA; 1QD72; 1QD89; 1QE14JAXXK; 1QE14JAXXL; 1QE14JAXXN; 1QE14JAXXP; 1QE53; 1QE56; 1QE58; 1QE59LAAD; 1QE59LAAG; 1QE59LAGX; 1QE59LAX7; 1QE72; 1QE76; 1QE80; 1QE82; 1QE84; 1QE87; 1QE89; 1QG14JAXXK; 1QG14JAXXL; 1QG14JAXXN; 1QG14JAXXP; 1QG53; 1QG56; 1QG59LAAD; 1QG59LAAG; 1QG59LAGX; 1QG59LAX7; 1QG78; 1QG80; 1QG87; 1QG89; 1QH80; 1QH87; 1QJ53; 1QJ58; 1QJ80; 1QJ87; 1QJ89LA; 1QM56; 1QM58; 1QM74; 1QM80; 1QM87; 1QM89; 1QM91; 1QN; 1QP51; 1QP52; 1QP72; 1QP73; 1QP87; 1QQ87; 1QQ89; 1QT87PB; 1QT87PK; 1QT87PNGX; 1QT87QZ; 1QT87QZAG; 1QT91; 1QZ89; 1QZ94DA; 1QZ94HA; 1QZ94LA; 1RB56LA; 1RB57DA; 1RB57LA; 1RB58; 1RB59; 1RB74; 1RB80; 1RB83; 1RB85; 1RB87; 1RB89; 1RD72; 1RD89; 1RF50DABJ; 1RF50DAGX; 1RF50DAKR; 1RF50DANR; 1RF50LABJ; 1RF50LAGX; 1RF50LAKR; 1RF50LANR; 1RF51; 1RF56; 1RF59DAAG; 1RF59DAGX; 1RF59LAAG; 1RF59LAGX; 1RF72; 1RF74; 1RF80; 1RF87; 1RF89; 1RM56DA; 1RM56LA; 1RM59DAGX; 1RM59LAGX; 1RM72CAGX; 1RM72DAGX; 1RM72LAGX; 1RM74; 1RM80DA; 1RM80LA; 1RM80LAXXA; 1RM80LAXXE; 1RM80LAXXF; 1RM80LAXXN; 1RM80LAXXQ; 1RM87DAAG; 1RM87DAAK; 1RM87DAGX; 1RM87LAAK; 1RM87LAGX; 1RM89; 1RM91; 1RN74LA; 1RN80DA; 1RN80LA; 1RN80LAFA; 1RN80LAXXA; 1RN80LAXXE; 1RN89LA; 1RN89LANRA; 1RN89LANRE; 1RN89LAXXA; 1RN89LAXXE; 1RN89LAXXQ; 1RS59DAGX; 1RS74; 1RS80CAXXA; 1RS80CAXXB; 1RS80CAXXE; 1RS80CAXXG; 1RS80CAXXN; 1RS80CAXXQ; 1RS80DA; 1RS80LA; 1RS80LAXXA; 1RS80LAXXB; 1RS80LAXXE; 1RS80LAXXG; 1RS80LAXXN; 1RS80LAXXQ; 1RS84; 1RS86; 1RS87; 1RS89; 1RW56LA; 1RW59; 1RW72; 1RW80; 1RW84; 1RW87; 1RW88; 1RW91; 1RW92; 1RY14JAXXK; 1RY14JAXXN; 1RY14JAXXP; 1RY56LA; 1RY59JAGX; 1RY80; 1RY87; 1RZ94DA; 1RZ94LA |
| Musculoskeletal (MSK) | 1VZ94LA; 1VZ70LA; 1VS80; 1VS72; 1VS58; 1VR80; 1VR72; 1VR58; 1VR57; 1VQ93; 1VQ91; 1VQ87; 1VQ83; 1VQ82; 1VQ80; 1VQ79; 1VQ74; 1VQ73LA; 1VQ58; 1VP89; 1VP87; 1VP80; 1VP74; 1VP73LA; 1VP72; 1VP53; 1VN; 1VM; 1VL; 1VK89; 1VK87; 1VK80; 1VG93; 1VG87; 1VG83; 1VG80; 1VG75; 1VG74; 1VG73LA; 1VG72LA; 1VG72DA; 1VG58DA; 1VG57; 1VG53; 1VE80; 1VE72; 1VE58; 1VD87; 1VD80; 1VD72; 1VD58; 1VD57; 1VC93; 1VC91; 1VC87; 1VC83; 1VC82; 1VC80; 1VC79; 1VC74; 1VC73LA; 1VC58; 1VA93; 1VA87; 1VA83; 1VA80; 1VA75; 1VA74; 1VA73LA; 1VA72LA; 1VA72DA; 1VA58DA; 1VA57; 1VA53; 1UV80; 1UV72; 1UV58; 1UU84; 1UU80; 1UU72; 1UU53; 1UT84; 1UT80; 1UT72LA; 1UT53; 1US80; 1US72; 1US58; 1UK93; 1UK87; 1UK80; 1UK75; 1UK74; 1UK73LA; 1UK72LA; 1UK53; 1UJ93; 1UJ87; 1UJ82; 1UJ80; 1UJ75; 1UJ74; 1UJ73LA; 1UJ71; 1UJ58; 1UG93; 1UG87; 1UG80; 1UG75; 1UG74; 1UG73LA; 1UG72LA; 1UG72JAHB; 1UG72JAAZ; 1UG57; 1UG53; 1UF93; 1UF87; 1UF84; 1UF82; 1UF80; 1UF79; 1UF74; 1UF73LA; 1UC89; 1UC87; 1UC82; 1UC80; 1UC79; 1C75; 1UC74; 1UC73LA; 1UC72; 1UC57; 1UC53; 1UB93; 1UB87; 1UB83; 1UB80; 1UB75; 1UB74; 1UB73LA; 1UB72LA; 1UB72DA; 1UB58; 1UB57; 1UB53; 1TZ94LA; 1TZ70LA; 1TV93; 1TV91; 1TV87; 1TV84; 1TV83; 1TV82; 1TV80; 1TV79; 1TV74; 1TV73LA; 1TV58; 1TS80; 1TS72; 1TS58; 1TQ80; 1TQ72; 1TQ58; 1TQ57; 1TM93; 1TM87; 1TM83; 1TM80; 1TM75; 1TM74; 1TM73LA; 1TM72LA; 1TM72DA; 1TM58; 1TM57; 1TM53; 1TK93; 1TK91; 1TK87; 1TK83; 1TK82; 1TK80; 1TK79; 1TK74; 1TK73LA; 1TK58; 1TH; 1TF80; 1TF72; 1TF58; 1TF57; 1TC80; 1TC72; 1TC57; 1TB87; 1TB80; 1TB74; 1TB72LA; 1TB72JAHB; 1TB72JAAZ; 1TB72DA; 1TA93; 1TA87; 1TA83; 1TA80; 1TA75; 1TA74; 1TA73LA; 1TA72LA; 1TA72DA; 1TA58; 1TA57; 1TA53; 1SY87; 1SY84; 1SY80; 1SY72; 1SY58; 1SY57; 1SY53; 1SW87; 1SW74; 1SQ93; 1SQ91; 1SQ87; 1SQ83; 1SQ80; 1SQ74; 1SQ58; 1SQ53; 1SN93; 1SN87; 1SN75; 1SN74; 1SN72; 1SN58; 1SM87; 1SM80; 1SM74; 1SM73LA; 1SL91; 1SL89; 1SL87; 1SL80; 1SL74; 1SL73LA; 1SL58; 1SK87; 1SK80; 1SK74; 1SK73LA; 1SG87; 1SG80; 1SG72WK; 1SG72WJ; 1SG58; 1SF89; 1SF87; 1SF80; 1SF75; 1SF74; 1SF73PF; 1SE89; 1SE87PF; 1SE59LAGX; 1SE53; 1SC89; 1SC87; 1SC80; 1SC75; 1SC74; 1SC72JAHB; 1SC72JAAZ; 1SA89; 1SA80; 1SA75; 1SA74 |
| Neurosurgery | 1AA53SZPL; 1AA80; 1AA87; 1AB86; 1AB87; 1AC50; 1AC52; 1AC53; 1AC59; 1AC87; 1AE53; 1AE59; 1AE85; 1AE87; 1AF59; 1AF87; 1AG87; 1AJ53; 1AJ87; 1AN53; 1AN56; 1AN59; 1AN70; 1AN73; 1AN87; 1AP52; 1AP53; 1AP59; 1AP72; 1AP87; 1AW59; 1AW72; 1AW87; 1AX52MESJ; 1AX52MQSJ; 1AX53; 1AX56; 1AX73; 1AX80; 1AX86; 1AX87; 1AZ94; 1BA53SZDV; 1BA59; 1BA72; 1BA80; 1BA87; 1BB53; 1BB58; 1BB59; 1BB72; 1BB80; 1BB87; 1BD58; 1BD59; 1BD72; 1BD80; 1BD87; 1BF; 1BG; 1BJ53; 1BJ59; 1BK59; 1BM; 1BN; 1BP; 1BQ; 1BS58; 1BS59; 1BS72; 1BS80; 1BS87; 1BT; 1BX53; 1BX59; 1BX72; 1BX80; 1BX87; 1BZ; 1JW50; 1JW51; 1JW57; 1JW59; 1JW76; 1JW89; 1JW86; 1JW87 |
| Ophthalmologic | 1CC58; 1CC59JAGX; 1CC80LAXXA; 1CC80LAXXK; 1CC80LAXXQ; 1CC84; 1CC85; 1CC87; 1CD52; 1CD53; 1CD80LAXXA; 1CD80LAXXK; 1CD87; 1CE56LA; 1CE56LALZ; 1CF59LAGX; 1CF87; 1CF91; 1CG59; 1CG71; 1CG76; 1CG87; 1CH59LAGX; 1CH72; 1CH80; 1CH87; 1CJ56; 1CJ59LAGX; 1CJ80; 1CJ87; 1CL53; 1CL56; 1CL59; 1CL87; 1CL89; 1CM59LA; 1CM89; 1CN59LAGX; 1CN59LAGY; 1CN72; 1CP53; 1CP56; 1CP80; 1CP87; 1CP89; 1CP91; 1CQ59LAGX; 1CQ72; 1CQ78; 1CQ80; 1CQ83; 1CQ87; 1CR80; 1CR87; 1CS56LA; 1CS56LALZ; 1CS59JAGX; 1CS72; 1CS80; 1CS84; 1CS87; 1CT51; 1CT59; 1CT80; 1CT87; 1CT89; 1CU50; 1CU51; 1CU56; 1CU57; 1CU59LAGX; 1CU72; 1CU76LA; 1CU76LANR; 1CU76ML; 1CU76MLNR; 1CU80; 1CU87; 1CU89; 1CV; 1CX56LA; 1CX59JAGX; 1CX72; 1CX74; 1CX78; 1CX80; 1CX84; 1CX87; 1CX88; 1CZ56LA; 1CZ70; 1CZ94HA; 1CZ94LA |
| Retroperitoneal | 1PB87; 1PB89; 1PC51LALV; 1PC56; 1PC59DAGX; 1PC59LAGX; 1PC80; 1PC82; 1PC83; 1PC86; 1PE50DABD; 1PE50DABF; 1PE50DABJ; 1PE56DA; 1PE56LA; 1PE59LAAG; 1PE59LAGX; 1PE76; 1PE77; 1PE80; 1PE82; 1PE87; 1PE89; 1PG50DABD; 1PG50DABF; 1PG50DABJ; 1PG50LABJ; 1PG52DA; 1PG56DA; 1PG56LA; 1PG57DAGX; 1PG57LAAM; 1PG57LAGX; 1PG59DAAG; 1PG59DAAS; 1PG59DAAT; 1PG59DAAZ; 1PG59DAGX; 1PG59KQAP; 1PG59KQAQ; 1PG59KQAR; 1PG59LAAG; 1PG59LAGX; 1PG72; 1PG74; 1PG76; 1PG77; 1PG80DA; 1PG80LA; 1PG80LAXXE; 1PG80LD; 1PG82; 1PG86; 1PG87; 1PG98; 1PL50LAGX; 1PL53; 1PL59LAAD; 1PL59LAAG; 1PL59LAAS; 1PL59LAAZ; 1PL59LAGX; 1PL72LA; 1PL72LAAG; 1PL74; 1PL80; 1PL87; 1PM56DA; 1PM56LA; 1PM57DAGX; 1PM57LAGX; 1PM58LA; 1PM59DAAG; 1PM59DAAS; 1PM59DAAT; 1PM59DAAZ; 1PM59DAGX; 1PM59DAX7; 1PM59KQAP; 1PM59KQAQ; 1PM59KQAR; 1PM72; 1PM77; 1PM79; 1PM80AF; 1PM80FJ; 1PM80LA; 1PM82; 1PM84; 1PM86; 1PM87LA; 1PM89LA; 1PM90; 1PM91; 1PM92 |
| Skin and Soft Tissue | 1MD87; 1MD89; 1MK87; 1MK89; 1MR50; 1MR51; 1MR52HA; 1MR59; 1MR76; 1MR80; 1MR87; 1MR91; 1MS50; 1MS51; 1MS52; 1MS59; 1MS76; 1MS80; 1MS87; 1MS91; 1SH56LA; 1SH59; 1SH87; 1SZ56LA; 1SZ59; 1SZ87; 1TX56LA; 1TX59LA; 1TX59LAGX; 1TX87; 1UY56LA; 1UY57; 1UY59; 1UY72; 1UY80; 1UY87; 1VX56LA; 1VX59LAGX; 1VX87; 1WV56LA; 1WV57; 1WV58; 1WV59LAGX; 1WV72; 1WV80; 1WV87; 1YA14JAXXK; 1YA14JAXXL; 1YA14JAXXN; 1YA14JAXXP; 1YA53; 1YA58; 1YA59JAGX; 1YA59JALV; 1YA80; 1YA83; 1YA87; 1YB14JAXXK; 1YB14JAXXL; 1YB14JAXXN; 1YB14JAXXP; 1YB53; 1YB58; 1YB59JAGX; 1YB74; 1YB80LAXXA; 1YB80LAXXB; 1YB80LAXXE; 1YB80LAXXF; 1YB87; 1YC14JAXXK; 1YC14JAXXL; 1YC14JAXXN; 1YC14JAXXP; 1YC58LAXXA; 1YC59JAGX; 1YC80LAXXA; 1YC80LAXXB; 1YC80LAXXE; 1YC87; 1YD14JAXXK; 1YD14JAXXL; 1YD14JAXXN; 1YD14JAXXP; 1YD59JAGX; 1YD80LAXXA; 1YD80LAXXB; 1YD80LAXXE; 1YD80LAXXF; 1YD87; 1YE14JAXXK; 1YE14JAXXL; 1YE14JAXXN; 1YE14JAXXP; 1YE59JAGX; 1YE74; 1YE78; 1YE79LAXXA; 1YE79LAXXL; 1YE79LAXXN; 1YE80LAXXA; 1YE80LAXXB; 1YE80LAXXE; 1YE87; 1YF14JAXXK; 1YF14JAXXL; 1YF14JAXXN; 1YF14JAXXP; 1YF53; 1YF57JACF; 1YF58; 1YF59JAGX; 1YF74; 1YF80LAXXA; 1YF80LAXXB; 1YF80LAXXE; 1YF80LAXXF; 1YF87; 1YG14JAXXK; 1YG14JAXXL; 1YG14JAXXN; 1YG14JAXXP; 1YG53; 1YG58; 1YG59JAGX; 1YG74; 1YG80LAXXA; 1YG80LAXXB; 1YG80LAXXE; 1YG80LAXXF; 1YG87; 1YR14JAXXK; 1YR14JAXXL; 1YR14JAXXN; 1YR14JAXXP; 1YR59JAGX; 1YR78LA; 1YR78LAAZ; 1YR80LAXXA; 1YR80LAXXB; 1YR80LAXXE; 1YR80LAXXF; 1YR87; 1YS14JAXXK; 1YS14JAXXL; 1YS14JAXXN; 1YS14JAXXP; 1YS53; 1YS58; 1YS59JAGX; 1YS74; 1YS78LA; 1YS78LAAZ; 1YS80; 1YS87; 1YT14JAXXK; 1YT14JAXXL; 1YT14JAXXN; 1YT14JAXXP; 1YT53; 1YT58; 1YT59JAGX; 1YT78HA; 1YT78LA; 1YT78LAAZ; 1YT80; 1YT87; 1YU14JAXXK; 1YU14JAXXL; 1YU14JAXXN; 1YU14JAXXP; 1YU53; 1YU58; 1YU59JAGX; 1YU80LAXXA; 1YU80LAXXB; 1YU80LAXXE; 1YU80LAXXF; 1YU80LAXXG; 1YU87; 1YV14JAXXK; 1YV14JAXXL; 1YV14JAXXN; 1YV14JAXXP; 1YV53; 1YV58; 1YV59JAGX; 1YV74; 1YV78LA; 1YV78LAAZ; 1YV80LAXXA; 1YV80LAXXB; 1YV80LAXXE; 1YV80LAXXF; 1YV87; 1YW14JAXXK; 1YW14JAXXL; 1YW14JAXXN; 1YW14JAXXP; 1YW53; 1YW58; 1YW59JAGX; 1YW80LAXXA; 1YW80LAXXB; 1YW80LAXXE; 1YW80LAXXF; 1YW87; 1YX80; 1YX87; 1YX89; 1YY14JAXXK; 1YY14JAXXL; 1YY14JAXXN; 1YY14JAXXP; 1YY80; 1YY87; 1YY87; 1YY89; 1YZ14JAXXK; 1YZ14JAXXL; 1YZ14JAXXN; 1YZ14JAXXP; 1YZ53; 1YZ58; 1YZ59JAGX; 1YZ59JALV; 1YZ78LA; 1YZ78LAAZ; 1YZ80LAXXA; 1YZ80LAXXB; 1YZ80LAXXE; 1YZ80LAXXF; 1YZ87; 1YZ94; 1YZ94LA |
| Retroperitoneal | 1PB87; 1PB89; 1PC51LALV; 1PC56; 1PC59DAGX; 1PC59LAGX; 1PC80; 1PC82; 1PC83; 1PC86; 1PE50DABD; 1PE50DABF; 1PE50DABJ; 1PE56DA; 1PE56LA; 1PE59LAAG; 1PE59LAGX; 1PE76; 1PE77; 1PE80; 1PE82; 1PE87; 1PE89; 1PG50DABD; 1PG50DABF; 1PG50DABJ; 1PG50LABJ; 1PG52DA; 1PG56DA; 1PG56LA; 1PG57DAGX; 1PG57LAAM; 1PG57LAGX; 1PG59DAAG; 1PG59DAAS; 1PG59DAAT; 1PG59DAAZ; 1PG59DAGX; 1PG59KQAP; 1PG59KQAQ; 1PG59KQAR; 1PG59LAAG; 1PG59LAGX; 1PG72; 1PG74; 1PG76; 1PG77; 1PG80DA; 1PG80LA; 1PG80LAXXE; 1PG80LD; 1PG82; 1PG86; 1PG87; 1PG98; 1PL50LAGX; 1PL53; 1PL59LAAD; 1PL59LAAG; 1PL59LAAS; 1PL59LAAZ; 1PL59LAGX; 1PL72LA; 1PL72LAAG; 1PL74; 1PL80; 1PL87; 1PM56DA; 1PM56LA; 1PM57DAGX; 1PM57LAGX; 1PM58LA; 1PM59DAAG; 1PM59DAAS; 1PM59DAAT; 1PM59DAAZ; 1PM59DAGX; 1PM59DAX7; 1PM59KQAP; 1PM59KQAQ; 1PM59KQAR; 1PM72; 1PM77; 1PM79; 1PM80AF; 1PM80FJ; 1PM80LA; 1PM82; 1PM84; 1PM86; 1PM87LA; 1PM89LA; 1PM90; 1PM91; 1PM92 |
| Thoracic | 1GM50LA; 1GM56LA; 1GM80DAXXE; 1GM80LA; 1GM80LAXXE; 1GM80LAXXG; 1GM86; 1GM87; 1GN; 1GR; 1GT56; 1GT58; 1GT59; 1GT78; 1GT80; 1GT85; 1GT87; 1GT89; 1GT91; 1GV56; 1GV59DAGX; 1GV59DAZ9; 1GV59LAGX; 1GV76; 1GV80; 1GV87; 1GV89; 1GW56; 1GW59; 1GW87; 1GX78; 1GX80; 1GX86; 1GX87; 1GY56; 1GY70; 1GY72; 1GY86; 1GY94DA; 1GY94LA; 1ME87; 1ME89; 1MF87; 1MM; 1MN50; 1MN51; 1MN59; 1MN74; 1MN76; 1MN77; 1MN80; 1MN87; 1NA56DB; 1NA56DBXXF; 1NA56DBXXG; 1NA56EZ; 1NA56EZXXG; 1NA56FA; 1NA56FAXXF; 1NA56FAXXG; 1NA56LB; 1NA56LBXXF; 1NA56LBXXG; 1NA56LP; 1NA56LPXXF; 1NA56LPXXG; 1NA56QB; 1NA56QBXXF; 1NA56QBXXG; 1NA56QFXXF; 1NA56QFXXG; 1NA72; 1NA74; 1NA76; 1NA77; 1NA80; 1NA82; 1NA84; 1NA86; 1NA87; 1NA88; 1NA89; 1NA90; 1NA91; 1NA92 |
| Vascular | 1ID57; 1ID76; 1ID80; 1ID82; 1ID86; 1ID87; 1JD53; 1JD59; 1JD89; 1JE50; 1JE51; 1JE57; 1JE58; 1JE59; 1JE76; 1JE80; 1JE87; 1JJ50; 1JJ51; 1JJ57; 1JJ58; 1JJ76; 1JJ80; 1JJ83; 1JJ87; 1JK50; 1JK51; 1JK57; 1JK58; 1JK76; 1JK80; 1JK87; 1JL50; 1JL51; 1JL57; 1JL58; 1JL80; 1JL87; 1JM50; 1JM51; 1JM57; 1JM58; 1JM76; 1JM80; 1JM82; 1JM87; 1JQ50; 1JQ51; 1JQ57; 1JQ80; 1JQ87; 1JT50; 1JT51; 1JT57; 1JT58; 1JT80; 1JT87; 1JU; 1JY50; 1JY51; 1JY57; 1JY76; 1JY80; 1JY87; 1KA50; 1KA53; 1KA57; 1KA58; 1KA76; 1KA80; 1KA82; 1KA87; 1KE50; 1KE51; 1KE57; 1KE58; 1KE76; 1KE80; 1KE87; 1KG50; 1KG51; 1KG57; 1KG58; 1KG76; 1KG80; 1KG82; 1KG87; 1KQ; 1KR34; 1KR50; 1KR51; 1KR53; 1KR57; 1KR58; 1KR59; 1KR76; 1KR78; 1KR80; 1KR83; 1KR87; 1KT50; 1KT51; 1KT58; 1KT76; 1KT80; 1KT82; 1KT87; 1KV53; 1KV80; 1KX53; 1KX80; 1KY*; 1KZ  *1KY with the exception of arteriovenous fistula creation (in separate category) |

**Supplementary Table 3.** Algorithms of ICD-9 and 10 codes used to define primary and secondary outcomes

| **Outcomes** | **ICD-9-CM diagnostic codes** | **ICD-10-CA diagnostic codes** | **Validation or Summary Studies** | **Data Source** |
| --- | --- | --- | --- | --- |
| Death | Any cause of death from Vital Statistics file | Any cause of death from Vital Statistics file | N/A | Vital Statistics |
| Acute Myocardial Infarction | 410 | I21 I22 | ^2,3^ | Hospitalization data file |
| CV Mortality - composite of: | | | | |
| CHD Death | 410 to 414, 419.2 (from death certificates) | I20 to I25 (from death certificates) | ^4^ | Vital Statistics |
| Ventricular Arrhythmia and Sudden Cardiac Death | Hospitalization data: 427.1, 427.4, 427.41, 427.42, 427.5, 427.9, 798, 798.1, 798.2 (from inpatient data)  [converted to ICD10: I472, I4901 I4902 I469 I499 R99] | None | ^5^ | Hospitalization data files |
|  | Death Data: 401.9, 402.9, 410, 411, 414.0, 414.8, 414.9, 425.4, 427.1, 427.4, 427.5, 427.9, 429.2, 429.9, 440.9, 798.9 (from death certificates)  [converted to ICD10: I10 I119 I110 I2109 I241 I200 I240 I248 I2510 I255 I2589 I259 I425 I428 I472 I4901 I4902 I469 I499 I2510 I519 I7090 I7091 R99] | None | ^5^ | Vital Statistics |

CV, cardiovascular; CHD, coronary heart disease; ICD, International Classification of Diseases (9^th^ and 10^th^ revision). Both most responsible diagnosis and post-admission diagnosis code types were included in these outcome ascertainment algorithms.

**Supplementary Table 4.** Variables that were considered as potential confounders of the association of interest, along with source of data and ICD-9/10 algorithms if applicable

| **Data Element** | **Data Element Source** | **Data element Description** |
| --- | --- | --- |
| ***Demographic Variables*** | | |
| Age | Alberta Health Registry | In years, calculated as index date minus date of birth; continuous. |
| Sex | Alberta Health Registry | Dichotomous Male and Female |
| Social Deprivation Index^6^ | Alberta Health Registry | Stratified into quintiles of social deprivation |
| ***Surgical Variables*** | | |
| Category of surgery | Hospitalization (ICD-10-CA) codes | Categorized into 14 surgical groups per CCI codes as outlined in Supplementary Table 2. |
| ***Comorbidities (all defined with unrestricted lookback)^7^*** | | |
| Cancer (any) | Hospitalization (ICD-10-CA) and Physician claims (Enhanced ICD-9-CM) | ICD-10: C00.x–C26.x, C30.x–C34.x, C37.x–  C41.x, C43.x, C45.x–C58.x, C60.x–C76.x, C81.x–C85.x, C88.x, C90.x–C97.x; ICD-9: 140.x–172.x, 174.x–195.8, 200.x–208.x, 238.6  ICD-10: C77.x-C80.x; ICD-9: 196.x-199.x |
| Cerebrovascular disease | Hospitalization (ICD-10-CA) and Physician claims (Enhanced ICD-9-CM) | ICD-10: G45.x, G46.x, H34.0, 160.x-169.x; ICD-9: 362.34, 430.x-438.x |
| Congestive Heart Failure | Hospitalization (ICD-10-CA) and Physician claims (Enhanced ICD-9-CM) | ICD-10: I09.9, I11.0, I13.0, I13.2, I25.5, I42.0, I42.5-I42.9, I43.x, I50.x, P29.0; ICD-9: 398.91, 402.01, 402.11, 402.91, 404.01, 404.03, 404.11, 404.13, 404.91, 404.93, 425.4–425.9, 428.x |
| Chronic pulmonary disease | Hospitalization (ICD-10-CA) and Physician claims (Enhanced ICD-9-CM) | ICD-10: I27.8, I27.9, J40.x–J47.x, J60.x–J67.x,  J68.4, J70.1, J70.3; ICD-9: 416.8, 416.9, 490.x–505.x, 506.4, 508.1, 508.8 |
| Dementia | Hospitalization (ICD-10-CA) and Physician claims (Enhanced ICD-9-CM) | ICD-10: F00.x–F03.x, F05.1, G30.x, G31.1 290.x; ICD-9: 294.1, 331.2 |
| Diabetes with and without complications | Hospitalization (ICD-10-CA) and Physician claims (Enhanced ICD-9-CM) | ICD-10: E10.0, E10.1, E10.6, E10.8, E10.9, E11.0, E11.1, E11.6, E11.8, E11.9, E12.0, E12.1, E12.6, E12.8, E12.9, E13.0, E13.1, E13.6, E13.8, E13.9, E14.0, E14.1, E14.6, E14.8, E14.9; ICD-9: 250.0–250.3, 250.8, 250.9  ICD-10: E10.2–E10.5, E10.7, E11.2–E11.5,  E11.7, E12.2–E12.5, E12.7, E13.2–  E13.5, E13.7, E14.2–E14.5, E14.7; ICD-9: 250.4-250.7 |
| Hypertension | Hospitalization (ICD-10-CA) and Physician claims (Enhanced ICD-9-CM) | ICD-10: I10-I13, I15  ICD-9: 401-405 |
| Liver disease (mild and moderate/severe) | Hospitalization (ICD-10-CA) and Physician claims (Enhanced ICD-9-CM) | ICD-10: B18.x, K70.0–K70.3, K70.9,  K71.3–K71.5, K71.7, K73.x, K74.x,  K76.0, K76.2–K76.4, K76.8, K76.9,  Z94.4; ICD-9: 070.22, 070.23, 070.32, 070.33,  070.44, 070.54, 070.6, 070.9,  570.x, 571.x, 573.3, 573.4,  573.8, 573.9, V42.7  ICD-10: I85.0, I85.9, I86.4, I98.2, K70.4,  K71.1, K72.1, K72.9, K76.5, K76.6,  K76.7; ICD-9: 456.0–456.2, 572.2–572.8 |
| Myocardial Infarction | Hospitalization (ICD-10-CA) and Physician claims (Enhanced ICD-9-CM) | ICD-10: I21.x, I22.x, I25.2; ICD-9:410.x, 412.x |
| Obesity | Physician claims (Enhanced ICD-9-CM) | Procedural modifier codes for billing: BMI, BMIABD, BMIANE, BMIANT, BMISRG, BMI2AN, BMIPRO. ^8^ |
| Paraplegia or hemiplegia | Hospitalization (ICD-10-CA) and Physician claims (Enhanced ICD-9-CM) | ICD-10: G04.1, G11.4, G80.1, G80.2, G81.x,  G82.x, G83.0–G83.4, G83.9; ICD-9: 334.1, 342.x, 343.x, 344.0–344.6, 344.9 |
| Peptic Ulcer disease | Hospitalization (ICD-10-CA) and Physician claims (Enhanced ICD-9-CM) | ICD-10: K25.x-K28.x; ICD-9: 531.x-534.x |
| Peripheral Vascular disease | Hospitalization (ICD-10-CA) and Physician claims (Enhanced ICD-9-CM) | ICD-10: I70.x, I71.x, I73.1, I73.8, I73.9, I77.1,  I79.0, I79.2, K55.1, K55.8, K55.9, Z95.8, Z95.9; ICD-9: 093.0, 437.3, 440.x, 441.x, 443.1–443.9, 47.1, 557.1, 557.9, V43.4 |
| Rheumatologic disease | Hospitalization (ICD-10-CA) and Physician claims (Enhanced ICD-9-CM) | ICD-10: M05.x, M06.x, M31.5, M32.x–M34.x,  M35.1, M35.3, M36.0; ICD-9: 446.5, 710.0–710.4, 714.0– 714.2, 714.8, 725.x |
| ***Kidney Failure related variables*** | | |
| Kidney Failure Type | Renal registry records | Coded as hemodialysis, peritoneal dialysis, or non-dialysis |
| Vintage | Renal registry records | In years, how long has the patient been in receipt of chronic dialysis therapy (at least 90 days) prior to the surgical procedure (defined as date of procedure minus first dialysis treatment pre-operatively). Patients must have been receiving dialysis immediately before the surgery as well (i.e. had to be a “current” chronic dialysis patient) |
| ***Laboratory investigations*** | | |
| Hemoglobin (g/L) | Alberta Laboratory data | Most recent preoperative outpatient hemoglobin that was drawn prior to the procedure, i.e. date of hemoglobin measure minus admission date is ≥1 day, from outpatient lab, and within one year before the procedure itself. |
| Albumin (g/L) | Alberta Laboratory data | Most recent preoperative outpatient albumin drawn prior to the procedure, i.e. date of albumin measure minus admission date is ≥1 day, from outpatient lab, and within one year before the procedure itself. |
| Sodium (mmol/L) | Alberta Laboratory data | Most recent preoperative outpatient sodium drawn prior to the procedure, i.e. date of sodium measure minus admission date is ≥1 day, from outpatient lab, and within one year before the procedure itself. |
| ***Pre-procedure details*** | | |
| Urgent status | Hospitalization codes (from SEPI_ADMIT_CAT_Code variable) | Defined as having “urgent” admission code attributed with index hospitalization for procedure. |
| Past hospitalizations | Hospitalization codes (ICD-10-CA) | Categorical variable if patients have been admitted to an acute care hospital in the year prior to the index procedure date. |

**Supplementary Table 5.** Cohort Baseline Characteristics, stratified by procedure type

|  | **Total (n=3398)** |  | **Anorectal (n=34)** |  | **Breast (n=22)** | | **Dialysis Access (n=262)** | | **Head and Neck (n=192)** | | **Intra-abdominal (n=351)** | | **Kidney Transplant (n=923)** | | **Lower Urologic and Gynecologic (n=61)** | | **Musculoskeletal (n=627)** | | **Neurosurgery (n=21)** | | **Ophthalmology (n=105)** | | **Retroperitoneal (n=20)** | | **Skin and Soft Tissue (n=124)** | | **Thoracic (n=29)** | | **Vascular (n=627)** | |
| --- | --- | --- | --- | --- | --- | --- | --- | --- | --- | --- | --- | --- | --- | --- | --- | --- | --- | --- | --- | --- | --- | --- | --- | --- | --- | --- | --- | --- | --- | --- |
|  | No. | % | No. | % | No. | % | No. | % | No. | % | No. | % | No. | % | No. | % | No. | % | No. | % | No. | % | No. | % | No. | % | No. | % | No. | % |
| **Sex** |  |  |  |  |  |  |  |  |  |  |  |  |  |  |  |  |  |  |  |  |  |  |  |  |  |  |  |  |  |  |
| Male | 2072 | 61 | 18 | 52.9 | 2 | 9.1 | 154 | 58.8 | 107 | 55.7 | 212 | 60.4 | 628 | 68 | 31 | 50.8 | 366 | 58.4 | 13 | 61.9 | 63 | 60 | 15 | 75 | 74 | 59.7 | 17 | 58.6 | 372 | 59.3 |
| Female | 1326 | 39 | 16 | 47.1 | 20 | 90.9 | 108 | 41.2 | 85 | 44.3 | 139 | 39.6 | 295 | 32 | 30 | 49.2 | 261 | 41.6 | 8 | 38.1 | 42 | 40 | 5 | 25 | 50 | 40.3 | 12 | 41.4 | 255 | 40.7 |
| **Age** (years; IQR) | 61.5 (50.0-72.7) | | 66.2 (56.3-75.5) | | 71.0 (64.9-77.9) | | 64.0 (55.4-74.2) | | 57.8 (45.1-66.3) | | 63.7 (49.2-75.6) | | 52.0 (40.2-60.7) | | 56.1 (50.2-68.6) | | 70.5 (58.0-80.2) | | 63.7 (43.2-74.0) | | 62.9 (52.2-74.2) | | 64.9 (57.8-72.6) | | 63.1 (51.8-73.0) | | 61.4 (55.9-70.0) | | 67.3 (56.4-76.8) | |
| **CKD Type** |  |  |  |  |  |  |  |  |  |  |  |  |  |  |  |  |  |  |  |  |  |  |  |  |  |  |  |  |  |  |
| Non-dialysis | 903 | 26.6 | 13 | 38.2 | 7 | 31.8 | 148 | 56.5 | 35 | 18.2 | 104 | 29.6 | 216 | 23.4 | 25 | 41 | 157 | 25 | 6 | 28.6 | 27 | 25.7 | 13 | 65 | 24 | 19.4 | 9 | 31 | 119 | 19 |
| Hemodialysis | 1905 | 56.1 | 17 | 50 | 13 | 59.1 | 98 | 37.4 | 114 | 59.4 | 189 | 53.8 | 452 | 49 | 25 | 41 | 367 | 58.5 | 11 | 52.4 | 65 | 61.9 | 6 | 30 | 87 | 70.2 | 15 | 51.7 | 446 | 71.1 |
| Peritoneal dialysis | 590 | 17.4 | 4 | 11.8 | 2 | 9.1 | 16 | 6.1 | 43 | 22.4 | 58 | 16.5 | 255 | 27.6 | 11 | 18 | 103 | 16.4 | 4 | 19 | 13 | 12.4 | 1 | 5 | 13 | 10.5 | 5 | 17.2 | 62 | 9.9 |
| **Dialysis vintage** |  |  |  |  |  |  |  |  |  |  |  |  |  |  |  |  |  |  |  |  |  |  |  |  |  |  |  |  |  |  |
| Non-dialysis | 903 | 26.6 | 13 | 38.2 | 7 | 31.8 | 148 | 56.5 | 35 | 18.2 | 104 | 29.6 | 216 | 23.4 | 25 | 41 | 157 | 25 | 6 | 28.6 | 27 | 25.7 | 13 | 65 | 24 | 19.4 | 9 | 31 | 119 | 19 |
| 0 to <2 years | 1087 | 32 | 9 | 26.5 | 8 | 36.4 | 67 | 25.6 | 45 | 23.4 | 116 | 33 | 312 | 33.8 | 15 | 24.6 | 194 | 30.9 | 10 | 47.6 | 45 | 42.9 | 5 | 25 | 47 | 37.9 | 8 | 27.6 | 206 | 32.9 |
| 2 or more years | 1408 | 41.4 | 12 | 35.3 | 7 | 31.8 | 47 | 17.9 | 112 | 58.3 | 131 | 37.3 | 395 | 42.8 | 21 | 34.4 | 276 | 44 | 5 | 23.8 | 33 | 31.4 | 2 | 10 | 53 | 42.7 | 12 | 41.4 | 302 | 48.2 |
| **Procedure Urgency** |  |  |  |  |  |  |  |  |  |  |  |  |  |  |  |  |  |  |  |  |  |  |  |  |  |  |  |  |  |  |
| Elective | 1463 | 43.1 | 16 | 47.1 | 19 | 86.4 | 114 | 43.5 | 146 | 76 | 94 | 26.8 | 595 | 64.5 | 52 | 85.2 | 158 | 25.2 | 5 | 23.8 | 69 | 65.7 | 10 | 50 | 21 | 16.9 | 12 | 41.4 | 152 | 24.2 |
| Urgent/Emergent | 1935 | 56.9 | 18 | 52.9 | 3 | 13.6 | 148 | 56.5 | 46 | 24 | 257 | 73.2 | 328 | 35.5 | 9 | 14.8 | 469 | 74.8 | 16 | 76.2 | 36 | 34.3 | 10 | 50 | 103 | 83.1 | 17 | 58.6 | 475 | 75.8 |
| **Hospitalized in prior year** | 1639 | 48.2 | 19 | 55.9 | 7 | 31.8 | 140 | 53.4 | 68 | 35.4 | 187 | 53.3 | 283 | 30.7 | 25 | 41 | 360 | 57.4 | 10 | 47.6 | 52 | 49.5 | 10 | 50 | 87 | 70.2 | 17 | 58.6 | 374 | 59.6 |
| **Comorbidities** |  |  |  |  |  |  |  |  |  |  |  |  |  |  |  |  |  |  |  |  |  |  |  |  |  |  |  |  |  |  |
| Cancer | 795 | 23.4 | 20 | 58.8 | 19 | 86.4 | 49 | 18.7 | 65 | 33.9 | 135 | 38.5 | 111 | 12 | 34 | 55.7 | 139 | 22.2 | 3 | 14.3 | 16 | 15.2 | 13 | 65 | 29 | 23.4 | 18 | 62.1 | 144 | 23 |
| Cerebrovascular Disease | 838 | 24.7 | 10 | 29.4 | 5 | 22.7 | 76 | 29 | 48 | 25 | 89 | 25.4 | 104 | 11.3 | 9 | 14.8 | 207 | 33 | 12 | 57.1 | 23 | 21.9 | 3 | 15 | 35 | 28.2 | 9 | 31 | 208 | 33.2 |
| Congestive Heart Failure | 1442 | 42.4 | 16 | 47.1 | 13 | 59.1 | 126 | 48.1 | 62 | 32.3 | 159 | 45.3 | 169 | 18.3 | 19 | 31.1 | 351 | 56 | 9 | 42.9 | 51 | 48.6 | 5 | 25 | 78 | 62.9 | 12 | 41.4 | 372 | 59.3 |
| COPD | 1652 | 48.6 | 20 | 58.8 | 12 | 54.5 | 140 | 53.4 | 96 | 50 | 190 | 54.1 | 334 | 36.2 | 30 | 49.2 | 346 | 55.2 | 10 | 47.6 | 50 | 47.6 | 8 | 40 | 68 | 54.8 | 20 | 69 | 328 | 52.3 |
| Dementia | 239 | 7 | 2 | 5.9 | 0 | 0 | 13 | 5 | 7 | 3.6 | 29 | 8.3 | 9 | 1 | 0 | 0 | 95 | 15.2 | 2 | 9.5 | 14 | 13.3 | 1 | 5 | 14 | 11.3 | 2 | 6.9 | 51 | 8.1 |
| Diabetes | 1774 | 52.2 | 14 | 41.2 | 11 | 50 | 151 | 57.6 | 77 | 40.1 | 209 | 59.5 | 268 | 29 | 23 | 37.7 | 392 | 62.5 | 11 | 52.4 | 90 | 85.7 | 6 | 30 | 99 | 79.8 | 10 | 34.5 | 413 | 65.9 |
| Hypertension | 3200 | 94.2 | 30 | 88.2 | 21 | 95.5 | 247 | 94.3 | 172 | 89.6 | 341 | 97.2 | 849 | 92 | 52 | 85.2 | 603 | 96.2 | 19 | 90.5 | 97 | 92.4 | 15 | 75 | 121 | 97.6 | 26 | 89.7 | 607 | 96.8 |
| Liver Disease | 377 | 11.1 | 3 | 8.8 | 2 | 9.1 | 27 | 10.3 | 21 | 10.9 | 59 | 16.8 | 79 | 8.6 | 5 | 8.2 | 79 | 12.6 | 0 | 0 | 8 | 7.6 | 2 | 10 | 18 | 14.5 | 4 | 13.8 | 70 | 11.2 |
| Myocardial Infarction | 1025 | 30.2 | 5 | 14.7 | 7 | 31.8 | 73 | 27.9 | 47 | 24.5 | 122 | 34.8 | 149 | 16.1 | 13 | 21.3 | 234 | 37.3 | 8 | 38.1 | 27 | 25.7 | 6 | 30 | 43 | 34.7 | 5 | 17.2 | 286 | 45.6 |
| Obesity | 498 | 14.7 | 4 | 11.8 | 2 | 9.1 | 40 | 15.3 | 37 | 19.3 | 53 | 15.1 | 102 | 11.1 | 13 | 21.3 | 93 | 14.8 | 5 | 23.8 | 18 | 17.1 | 3 | 15 | 29 | 23.4 | 4 | 13.8 | 95 | 15.2 |
| Paraplegia | 152 | 4.5 | 2 | 5.9 | 1 | 4.5 | 16 | 6.1 | 10 | 5.2 | 15 | 4.3 | 14 | 1.5 | 1 | 1.6 | 31 | 4.9 | 4 | 19 | 5 | 4.8 | 2 | 10 | 9 | 7.3 | 2 | 6.9 | 40 | 6.4 |
| Peptic Ulcer Disease | 614 | 18.1 | 4 | 11.8 | 5 | 22.7 | 47 | 17.9 | 29 | 15.1 | 87 | 24.8 | 94 | 10.2 | 9 | 14.8 | 147 | 23.4 | 4 | 19 | 17 | 16.2 | 2 | 10 | 26 | 21 | 3 | 10.3 | 140 | 22.3 |
| Peripheral Vascular Disease | 1508 | 44.4 | 14 | 41.2 | 7 | 31.8 | 96 | 36.6 | 75 | 39.1 | 143 | 40.7 | 246 | 26.7 | 15 | 24.6 | 321 | 51.2 | 9 | 42.9 | 38 | 36.2 | 6 | 30 | 84 | 67.7 | 9 | 31 | 445 | 71 |
| Rheumatologic Disease | 336 | 9.9 | 4 | 11.8 | 4 | 18.2 | 28 | 10.7 | 12 | 6.3 | 48 | 13.7 | 75 | 8.1 | 1 | 1.6 | 72 | 11.5 | 2 | 9.5 | 10 | 9.5 | 3 | 15 | 7 | 5.6 | 1 | 3.4 | 69 | 11 |
| **Social Deprivation Index** |  |  |  |  |  |  |  |  |  |  |  |  |  |  |  |  |  |  |  |  |  |  |  |  |  |  |  |  |  |  |
| Least deprived | 525 | 15.5 | 6 | 17.6 | 6 | 27.3 | 40 | 15.3 | 23 | 12 | 51 | 14.5 | 179 | 19.4 | 5 | 8.2 | 91 | 14.5 | 6 | 28.6 | 10 | 9.5 | 3 | 15 | 9 | 7.3 | 5 | 17.2 | 91 | 14.5 |
| 2 | 414 | 12.2 | 6 | 17.6 | 1 | 4.5 | 38 | 14.5 | 20 | 10.4 | 39 | 11.1 | 142 | 15.4 | 7 | 11.5 | 74 | 11.8 | 3 | 14.3 | 8 | 7.6 | 2 | 10 | 10 | 8.1 | 4 | 13.8 | 60 | 9.6 |
| 3 | 544 | 16 | 3 | 8.8 | 0 | 0 | 45 | 17.2 | 37 | 19.3 | 55 | 15.7 | 160 | 17.4 | 7 | 11.5 | 81 | 12.9 | 4 | 19 | 18 | 17.1 | 3 | 15 | 26 | 21 | 5 | 17.2 | 100 | 15.9 |
| 4 | 756 | 22.3 | 9 | 26.5 | 6 | 27.3 | 52 | 19.8 | 42 | 21.9 | 77 | 21.9 | 206 | 22.3 | 16 | 26.2 | 140 | 22.3 | 2 | 9.5 | 30 | 28.6 | 4 | 20 | 25 | 20.2 | 6 | 20.7 | 141 | 22.5 |
| Most deprived | 927 | 27.3 | 6 | 17.6 | 6 | 27.3 | 65 | 24.8 | 57 | 29.7 | 107 | 30.5 | 210 | 22.8 | 22 | 36.1 | 178 | 28.4 | 5 | 23.8 | 29 | 27.6 | 6 | 30 | 42 | 33.9 | 9 | 31 | 185 | 29.5 |
| Not defined | 231 | 6.8 | 4 | 11.8 | 3 | 13.6 | 22 | 8.4 | 13 | 6.8 | 22 | 6.3 | 25 | 2.7 | 4 | 6.6 | 63 | 10 | 1 | 4.8 | 10 | 9.5 | 2 | 10 | 12 | 9.7 | 0 | 0 | 50 | 8 |
| **Serum sodium (mmol/L)** | 137 (134-139) | | 138 (136-140) | | 137 (134-140) | | 137.5 (135-140) | | 137.5 (134-139) | | 137 (134-139) | | 138 (135-139) | | 136.5 (135-139) | | 137 (134-139) | | 138 (134-139) | | 138 (135.5-140) | | 139 (137-140) | | 135.5 (132-138) | | 137 (134-139) | | 137 (134-139) | |
| Missing | 615 | 18.1 | 8 | 23.5 | 3 | 13.6 | 42 | 16 | 24 | 12.5 | 65 | 18.5 | 129 | 14 | 9 | 14.8 | 133 | 21.2 | 7 | 33.3 | 17 | 16.2 | 2 | 10 | 22 | 17.7 | 3 | 10.3 | 151 | 24.1 |
| **Serum albumin (g/L)** | 36 (32-39) | | 34 (28-38) | | 36 (31-40) | | 35 (31.5-39) | | 36 (32-39) | | 35 (30-38) | | 38 (35-41) | | 37.5 (34-40) | | 34 (30-38) | | 35.5 (32-37) | | 36 (32-39) | | 39 (35-41) | | 33 (28-36) | | 34.5 (30-38) | | 34 (31-37) | |
| Missing | 654 | 19.2 | 9 | 26.5 | 3 | 13.6 | 50 | 19.1 | 26 | 13.5 | 70 | 19.9 | 129 | 14 | 11 | 18 | 146 | 23.3 | 7 | 33.3 | 19 | 18.1 | 2 | 10 | 25 | 20.2 | 3 | 10.3 | 154 | 24.6 |
| **Serum hemoglobin (g/L)** | 109 (98-118) | | 105 (95-115) | | 109 (102-113) | | 105.5 (96-117) | | 108 (98-119) | | 104 (92-115) | | 111 (103-121) | | 109 (101-118) | | 108 (98-116) | | 103 (94-109) | | 110 (102-120) | | 109 (100-122.5) | | 102.5 (90.5-115.5) | | 103 (93-109) | | 109 (98-118) | |
| Missing | 3 | 0.1 | 0 | 0 | 1 | 4.5 | 0 | 0 | 0 | 0 | 0 | 0 | 1 | 0.1 | 0 | 0 | 1 | 0.2 | 0 | 0 | 0 | 0 | 0 | 0 | 0 | 0 | 0 | 0 | 0 | 0 |

AMI, acute myocardial infarction; CKD, chronic kidney disease; COPD, chronic obstructive pulmonary disease; CV, cardiovascular; g/L, grams per litre; IQR, interquartile range; mmol/L, millimoles per litre; No., number.

**Supplementary Table 6.** Unadjusted Odds of AMI and Death for people with kidney failure undergoing major non-cardiac surgery, stratified by surgical procedure type

| **Surgical Category** | **Number of People with Surgeries** | **Number of AMI + Death Events** | **Unadjusted Odds Ratio of AMI and Death within 30 days (95%CI)** |
| --- | --- | --- | --- |
| Kidney Transplant | 923 | 19 | [Reference] |
| Ophthalmology | 105 | 2 | 0.9 (0.2, 4.0) |
| Dialysis Access | 262 | 9 | 1.7 (0.8, 3.8) |
| Lower Urologic and Gynecologic | 61 | 2 | 1.6 (0.4, 7.1) |
| Head and Neck | 192 | 10 | 2.6 (1.2, 5.7) |
| Breast | 22 | 1 | 2.3 (0.3, 17.7) |
| Thoracic | 29 | 2 | 3.5 (0.8, 15.9) |
| Vascular | 627 | 79 | 6.9 (4.1, 11.4) |
| Skin and Soft Tissue | 124 | 15 | 6.5 (3.2, 13.3) |
| Intra-abdominal | 351 | 41 | 6.3 (3.6, 11.0) |
| Musculoskeletal | 627 | 77 | 6.7 (4.0, 11.1) |
| Retroperitoneal | 20 | 2 | 5.3 (1.1, 24.4) |
| Anorectal | 34 | 5 | 8.2 (2.9, 23.5) |
| Neurosurgery | 21 | 8 | 29.3 (10.9, 78.9) |
| Number included in analysis | - | - | 3398 |
| Bayesian Information Criterion (BIC) | - | - | 1871.9 |
| McFadden’s Adjusted Pseudo-R2 | - | - | 0.072 |

AMI, Acute Myocardial Infarction; CI, confidence interval.

**Supplementary Table 7.** Top Causes of Perioperative Death for people with Kidney Failure

| **Cause of Death** | **Number** | **Proportion** |
| --- | --- | --- |
| Atherosclerotic heart disease of native coronary artery | 16 | 9.1 |
| Chronic renal failure | 13 | 7.4 |
| Unspecified diabetes complication (peripheral angiopathy, gangrene, vascular complication etc.) | 11 | 6.3 |
| Acute myocardial infarction unspecified | 9 | 5.1 |
| Unspecified diabetes with kidney disease | 7 | 4 |
| Unspecified diabetes without complication | 6 | 3.4 |
| Vascular disorder of intestine | 6 | 3.4 |
| Unspecified renal failure | 6 | 3.4 |
| Atherosclerotic cardiovascular disease | 4 | 2.3 |
| Peripheral vascular disease unspecified | 4 | 2.3 |

**REFERENCES**

1. Hemmelgarn BR, Clement F, Manns BJ, et al. Overview of the Alberta Kidney Disease Network. *BMC Nephrol.* 2009;10:30.

2. Austin PC, Daly PA, Tu JV. A multicenter study of the coding accuracy of hospital discharge administrative data for patients admitted to cardiac care units in Ontario. *Am Heart J.* 2002;144(2):290-296.

3. Smilowitz NR, Gupta N, Guo Y, Berger JS, Bangalore S. Perioperative acute myocardial infarction associated with non-cardiac surgery. *Eur Heart J.* 2017;38(31):2409-2417.

4. Roger VL, Go AS, Lloyd-Jones DM, et al. Heart disease and stroke statistics--2012 update: a report from the American Heart Association. *Circulation.* 2012;125(1):e2-e220.

5. Ye Y, Larrat EP, Caffrey AR. Algorithms used to identify ventricular arrhythmias and sudden cardiac death in retrospective studies: a systematic literature review. *Ther Adv Cardiovasc Dis.* 2018;12(2):39-51.

6. Pampalon R, Hamel D, Gamache P, Simpson A, Philibert MD. Validation of a deprivation index for public health: a complex exercise illustrated by the Quebec index. *Chronic Dis Inj Can.* 2014;34(1):12-22.

7. Tonelli M, Wiebe N, Fortin M, et al. Methods for identifying 30 chronic conditions: application to administrative data. *BMC Med Inform Decis Mak.* 2015;15:31.

8. Tonelli M, Wiebe N, Kovesdy CP, et al. Joint associations of obesity and estimated GFR with clinical outcomes: a population-based cohort study. *BMC Nephrology.* 2019;20(1):204.
